# Development of a Health Behavioral Digital Intervention for Patients With Hypertension Based on an Intelligent Health Promotion System and WeChat: Randomized Controlled Trial

**DOI:** 10.2196/53006

**Published:** 2024-04-05

**Authors:** Ting Sun, Xuejie Xu, Zenghui Ding, Hui Xie, Linlin Ma, Jing Zhang, Yuxin Xia, Guoli Zhang, Zuchang Ma

**Affiliations:** 1 Graduate School University of Science and Technology of China Hefei China; 2 Hefei Institutes of Physical Science Chinese Academy of Sciences Hefei China; 3 School of Nursing Bengbu Medical University Bengbu China

**Keywords:** adherence, hypertension, health behavior, mHealth, digital health

## Abstract

**Background:**

The effectiveness of timely medication, physical activity (PA), a healthy diet, and blood pressure (BP) monitoring for promoting health outcomes and behavioral changes among patients with hypertension is supported by a substantial amount of literature, with “adherence” playing a pivotal role. Nevertheless, there is a lack of consistent evidence regarding whether digital interventions can improve adherence to healthy behaviors among individuals with hypertension.

**Objective:**

The aim was to develop a health behavioral digital intervention for hypertensive patients (HBDIHP) based on an intelligent health promotion system and WeChat following the behavior change wheel (BCW) theory and digital micro-intervention care (DMIC) model and assess its efficacy in controlling BP and improving healthy behavior adherence.

**Methods:**

A 2-arm, randomized trial design was used. We randomly assigned 68 individuals aged >60 years with hypertension in a 1:1 ratio to either the control or experimental group. The digital intervention was established through the following steps: (1) developing digital health education materials focused on adherence to exercise prescriptions, Dietary Approaches to Stop Hypertension (DASH), prescribed medication, and monitoring of BP; (2) using the BCW theory to select behavior change techniques; (3) constructing the intervention's logic following the guidelines of the DMIC model; (4) creating an intervention manual including the aforementioned elements. Prior to the experiment, participants underwent physical examinations at the community health service center's intelligent health cabin and received intelligent personalized health recommendations. The experimental group underwent a 12-week behavior intervention via WeChat, while the control group received routine health education and a self-management manual. The primary outcomes included BP and adherence indicators. Data analysis was performed using SPSS, with independent sample *t* tests, chi-square tests, paired *t* tests, and McNemar tests. A *P* value <.05 was considered statistically significant.

**Results:**

The final analysis included 54 participants with a mean age of 67.24 (SD 4.19) years (n=23 experimental group, n=31 control group). The experimental group had improvements in systolic BP (–7.36 mm Hg, *P*=.002), exercise time (856.35 metabolic equivalent [MET]-min/week, *P*<.001), medication adherence (0.56, *P*=.001), BP monitoring frequency (*P*=.02), and learning performance (3.23, *P*<.001). Both groups experienced weight reduction (experimental: 1.2 kg, *P*=.002; control: 1.11 kg, *P*=.009) after the intervention. The diet types and quantities for both groups (*P*<.001) as well as the subendocardial viability ratio (0.16, *P*=.01) showed significant improvement. However, there were no statistically significant changes in other health outcomes.

**Conclusions:**

The observations suggest our program may have enhanced specific health outcomes and adherence to health behaviors in older adults with hypertension. However, a longer-term, larger-scale trial is necessary to validate the effectiveness.

**Trial Registration:**

Chinese Clinical Trial Registry ChiCTR2200062643; https://www.chictr.org.cn/showprojEN.html?proj=172782

**International Registered Report Identifier (IRRID):**

RR2-10.2196/46883

## Introduction

### Hypertension and Health Behavior Interventions

Hypertension, as a high-prevalence chronic disease, has become an important risk factor for many diseases (eg, stroke, renal disease) and a major contributor to the global burden of disease [1,2]. Approximately one-third of older adults with hypertension fail to achieve their blood pressure (BP) control goals [3]. The reasons for the low rate of hypertension control are related to high-risk lifestyles such as poor dietary habits and low levels of physical activity (PA).

Relevant studies have shown that adherence to recommended health behaviors can significantly reduce systolic blood pressure (SBP) by an average of 4.0 mm Hg to 5.6 mm Hg and diastolic blood pressure (DBP) by an average of 4.1 mm Hg to 5.3 mm Hg in individuals with hypertension [4]. Engaging in a wide range of exercise training can lead to average reductions of 4.08 mm Hg to 8.24 mm Hg in SBP and 2.5 mm Hg to 4.0 mm Hg in DBP [5]. Regular BP monitoring behaviors result in average reductions of 2.53 mm Hg to 4.7 mm Hg in SBP and 1.45 mm Hg to 2.4 mm Hg in DBP [6,7]. Adherence to Dietary Approaches to Stop Hypertension (DASH) not only reduces SBP and DBP by approximately 5.5 mm Hg and 3 mm Hg, respectively, but also reduces the chance of developing hypertension by 26% [8,9]. High medication adherence, combined with comprehensive interventions like diet and exercise management, leads to improved BP control [10,11]. It is evident that comprehensive health behavior interventions can achieve effective BP control, with adherence playing a pivotal role.

In recent years, several studies exploring intelligent health promotion systems that incorporate advanced technologies like artificial intelligence, wearable devices, and mobile communication have consistently shown their efficacy in managing chronic diseases [12-14]. In addition, numerous studies support that independent mobile health (mHealth) apps play a vital role in community-based patient management [10,12,14-16]. WeChat, China's predominant social communication mobile app, serves as one platform for mHealth interventions. It boasts a staggering daily user count of up to 902 million people and over 1 billion monthly active users spanning all age groups [17]. Several studies have indicated that mHealth-based interventions can enhance health outcomes, quality of life, and self-care among patients with chronic diseases [18,19]. BP monitoring adherence (BPMA), dietary habits, and self-efficacy behaviors of patients with hypertension have been somewhat improved by these interventions based on mHealth [20-22]. However, other studies have shown no significant change or little improvement in BP, medicine, exercise, and DASH adherence [20,23-25].

### Theoretical Framework

**Figure 1:**
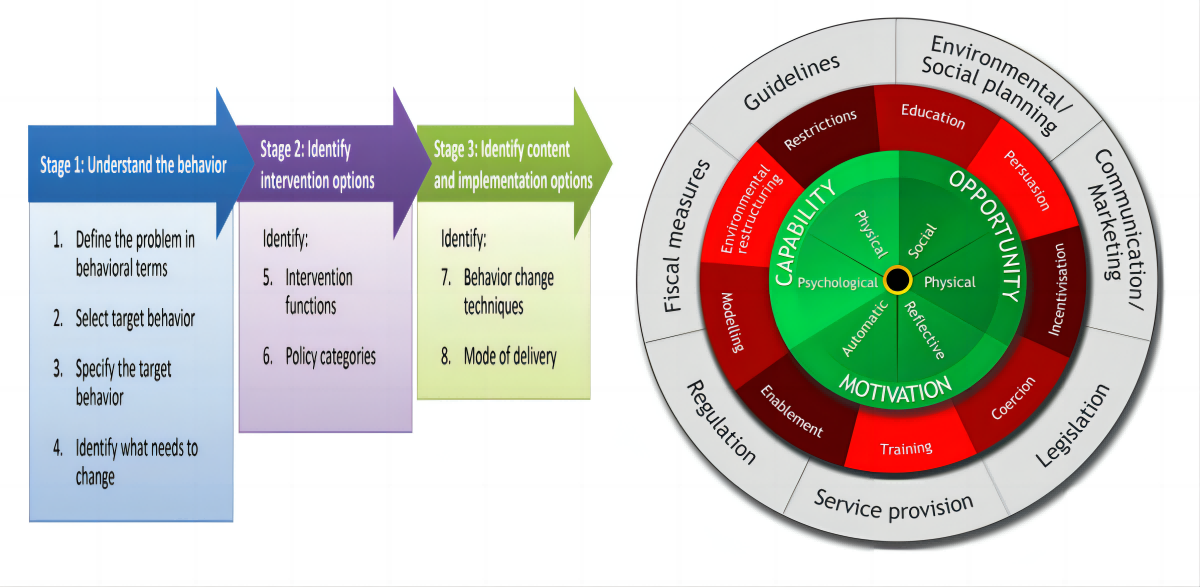
Behavior change wheel.

The behavior change wheel (BCW), which integrates 19 relevant theoretical frameworks for behavior change, was first proposed by Michie et al in 2011 [26]. As Figure 1 illustrates, it consists of 3 tiers. The inner tier is the Capability, Opportunity, Motivation-Behavior (COM-B) model, which is used to identify barriers to intervening in the target behavior. The second tier comprises the following 9 intervention categories intended to tackle identified behavioral obstacles: education, persuasion, motivation, coercion, training, restriction, environmental restructuring, demonstration, and empowerment. The outermost tier encompasses 7 policy categories (eg, regulation and legislation) that aid in the implementation of macrolevel interventions [27]. This theory consists of 3 steps: understanding the behavior, identifying intervention options, and identifying content and implementing the options (ie, behavior change techniques [BCTs]). Michie et al [28], along with other scholars, developed “The behavior change technique taxonomy of 93 hierarchically clustered techniques,” which contains 93 BCTs and provides names, definitions, and examples. In the final step, researchers can select the necessary BCTs from this taxonomy list.

Scholars have applied this theory to community health promotion, health care management, and nursing care, resulting in positive outcomes [29-32]. Additionally, some researchers have extended its application to digital and mHealth interventions [33]. The effectiveness of this lies in its ability to aid interveners in the systematic and scientific identification of intervention functions and specific BCTs for behavior change problems. Despite this, the theory does not provide much insight into the components of the intervention. The BCW can be supplemented by the digital micro-intervention care (DMIC) model [25,34].

In 2020, the DMIC model proposed by Baumel and colleagues [34] provided a reference paradigm. This theoretical model promotes shorter, more focused interventions, known as micro-interventions. They can be highly focused on implementation in people's daily lives to help intervention recipients achieve desired short-term goals (the basis for achieving long-term goals). The DMIC comprises the following 3 core concepts: events (in-the-moment attempts at change or impact toward the overall target of the intervention), decision rules (guiding which events are deployed and when), and proximal assessments (assessing the impact of the event). Events are similar to specific BCTs, while decision rules deploy events in a meaningful way based on time, user status, or environmental information, allowing interveners to dynamically adjust the content of micro-interventions. Overall outcome assessment, proximal event outcome assessment, and assessment of user participation in the micro-intervention are 3 types of impact assessment for digital micro- interventions. Based on the results of the overall and proximal assessments and the continuous recording of the user engagement experience (ie, measuring the quality of attention, engagement, and immersion during the use of the program), it is possible to identify individuals with low levels of engagement and later search for the causes, modify the intervention decision rules, and re-engage the user. This theoretical framework advocates for interventions that aim to achieve specific objectives through in-the-moment intervention elements. These elements may not be directly tied to the attainment of a broader clinical goal [34]. Each intervention in every event represents an immediate attempt to modify or influence the overall goal of the intervention. This implies that, in order to achieve a clinical objective, interventions should be broken down into numerous small steps and regulated through proximal assessments, ultimately leading to the attainment of the overall outcome.

### Goal of This Study

Therefore, our objective was to develop a health behavioral digital intervention for hypertensive patients (HBDIHP) based on BCW and DMIC and assess the effectiveness of this program in 2 groups after 3 months of intervention. This program involved exercise, diet, BP monitoring, and medication adherence intervention strategies. Consequently, we aimed to assess the effectiveness of this approach for enhancing outcomes for older adults with hypertension.

## Methods

### Development of HBDIHP

#### Community-Oriented Intelligent Health Promotion System

**Figure 2:**
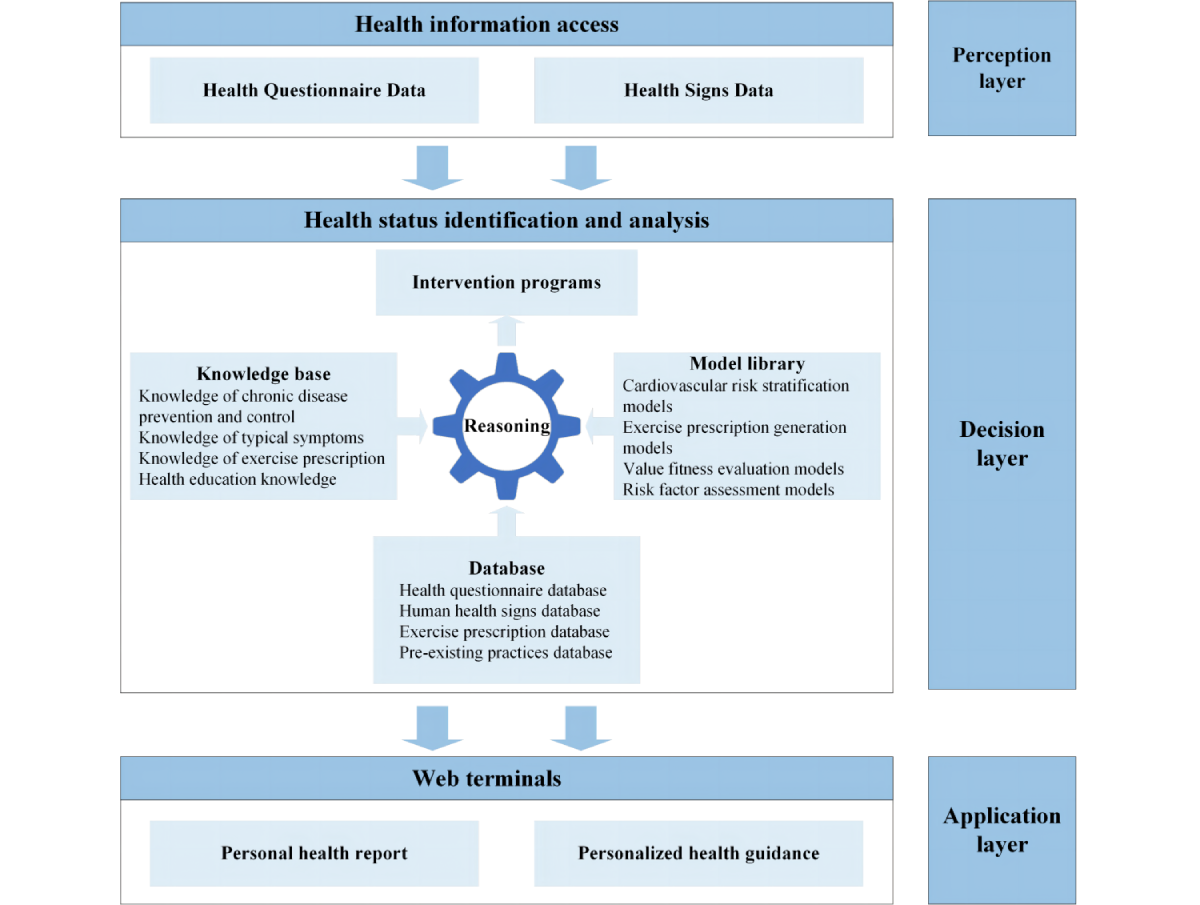
Intelligent health promotion system architecture diagram.

The Intelligent Health Promotion System is a cloud platform–based system that leverages health sign data and health questionnaire responses to generate intelligent health reports, personalized exercise prescriptions, and other tailored health recommendations. It also tracks and monitors individual health data. This system comprises the following 3 main layers: perception layer, decision layer, and application layer (see Figure 2).

**Figure 3:**
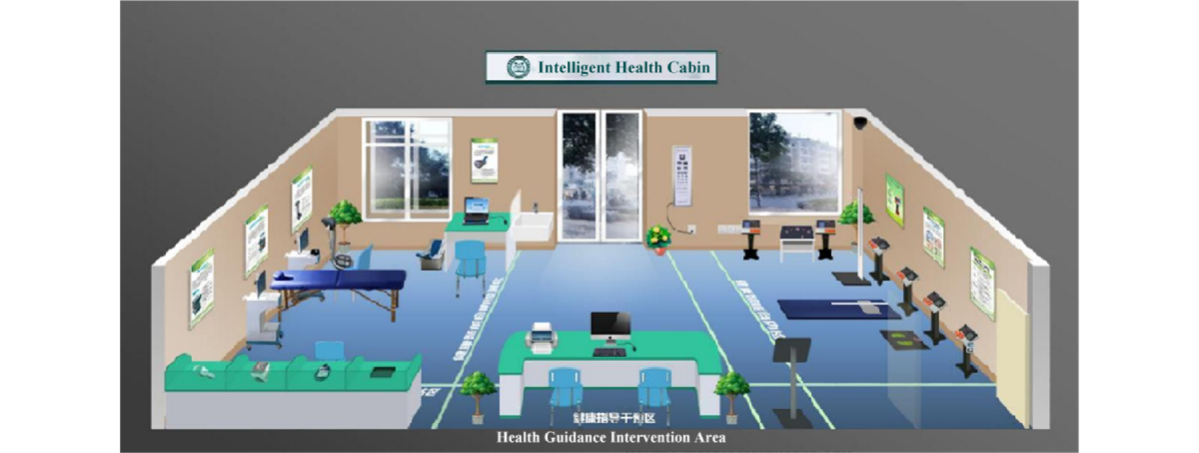
Intelligent Health Cabin.

This system is installed in the Intelligent Health Cabin at community health service centers (see Figure 3). It currently supports a range of connected instruments, including cardiovascular function monitors, arteriosclerosis detectors, body composition monitors, bone densitometers, and physical fitness detectors. After completing assessments with these instruments, the system sends those collected data to its central cloud platform database. Participants need to continue to fill out various questionnaires, such as chronic disease history questionnaires, medication profiles, and family medical history surveys. The system then uses both instrument data and questionnaire responses to activate the intelligent decision-making and inference engine, which generates comprehensive reports. These reports provide a comprehensive evaluation of an individual's health status and offer personalized, evidence-based recommendations, namely personalized intervention plans.

#### Assessment of Health Outcomes and Risk Prediction

The comprehensive report provides an overview of participants’ health, identifying health issues in areas such as the cardiovascular system, lipid metabolism, musculoskeletal system, lifestyle, and physical fitness, while explaining the meaning of abnormal indicators. In the cardiovascular assessment, the system not only evaluates participants’ cardiovascular systems based on instrument results but also predicts participants’ heart age and the risk of cardiovascular disease using a machine learning model.

#### Personalized Health Advice

**Figure 4:**
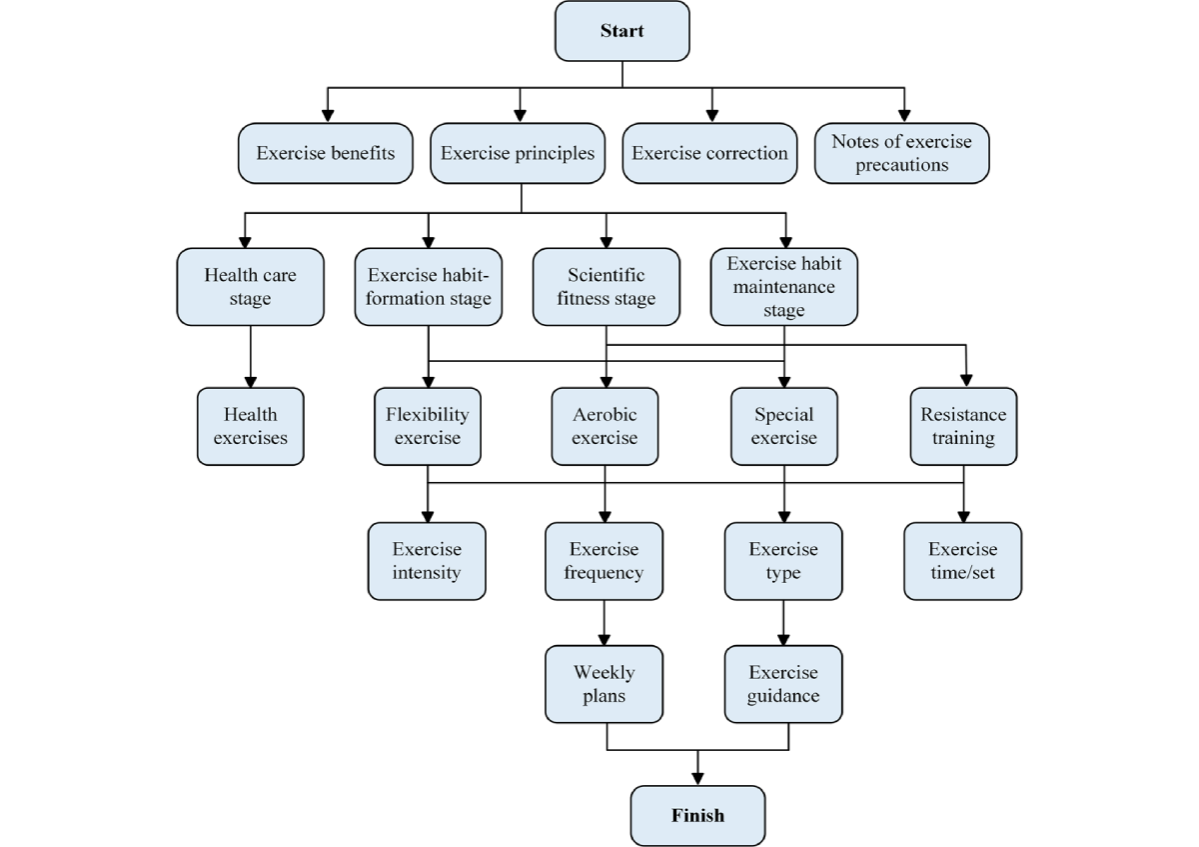
Exercise prescription generation flowchart.

**Figure 5:**
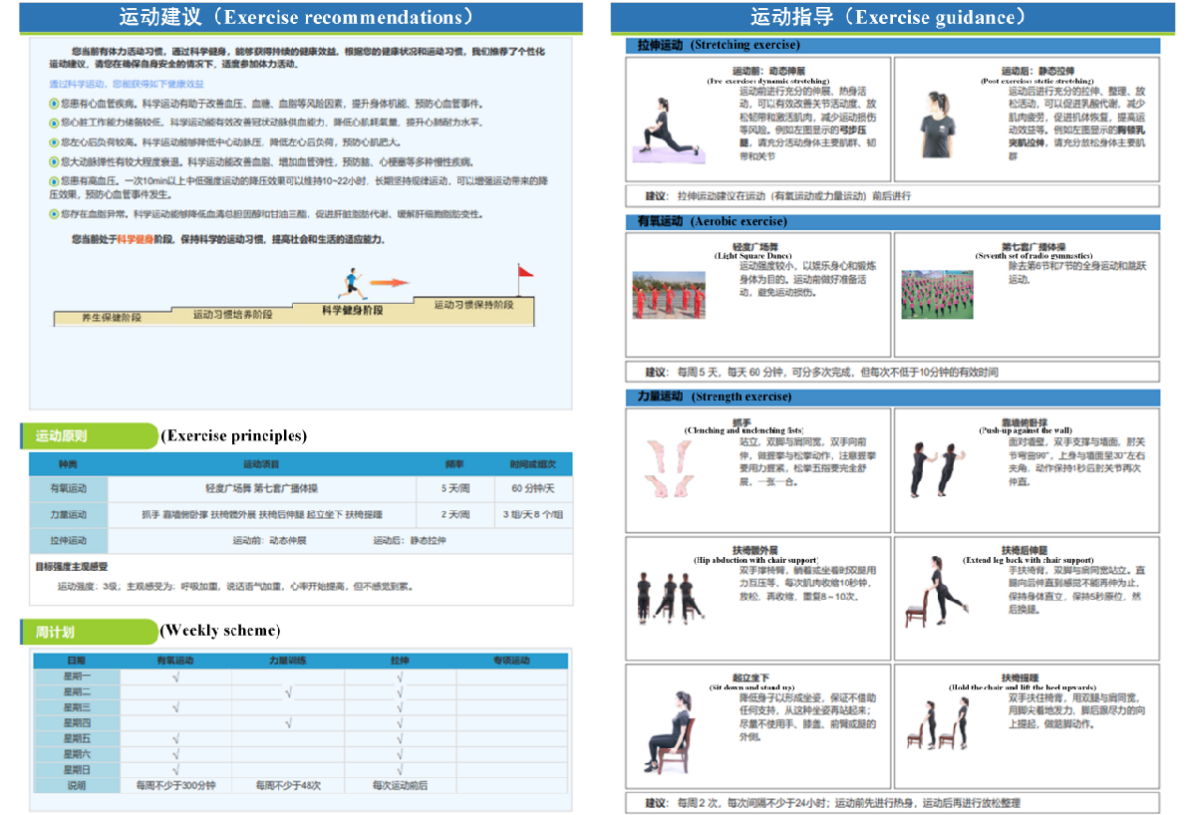
Personalized exercise prescription.

The comprehensive report offers personalized health advice to each resident, which includes exercise prescriptions, dietary recommendations, and suggestions for behavior correction. The exercise prescription, based on the design principles of the American College of Sports Medicine guidelines (frequency, intensity, type, time, volume, and progression [FITT-VP]) combined with the Transtheoretical Model, delineates intervention plans tailored to the health care stage, exercise habit formation stage, scientific fitness stage, and exercise habit maintenance stage. After being processed by the intelligent decision module, the system generates exercise prescriptions customized for individual residents (see Figure 4), considering the exclusion of exercise contraindications. These prescriptions encompass exercise recommendations, principles, weekly plans, exercise correction, precautions, and exercise guidance (see Figure 5) [13].

**Figure 6:**
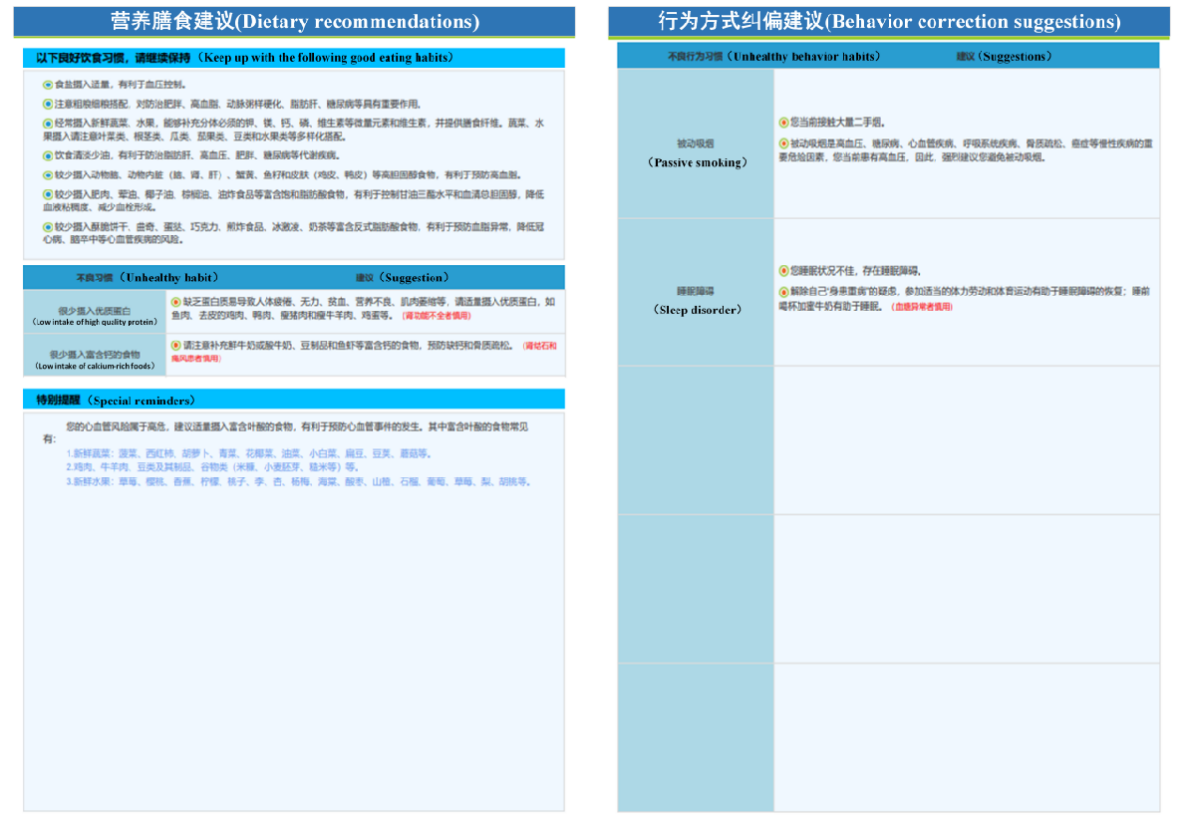
Dietary recommendations and behavior correction suggestions.

A dietary guidance and behavior correction database was constructed using technical strategies such as expert systems and knowledge graphs. In practice, the system provides dietary recommendations and behavior correction suggestions (eg, health advice for sedentary individuals to change their unhealthy habits) based on diet-related questionnaires, medical history collection, and physical examination results (see Figure 6).

### Development of the Health Behavioral Digital Intervention

A multidisciplinary working group for digital health intervention strategies was established during the entire intervention program design process. This group included 3 patients with hypertension, 1 clinical expert in hypertension, 1 management expert, and 1 behavioral psychology expert. Group-focused interviews were carried out at every stage of development. At this stage, experts evaluated intervention strategies using their professional knowledge, while patients with hypertension assessed the acceptability and utility of the interventions from their perspective. After finalizing the intervention scheme, experts who were not involved in the development were invited to assess its structural validity.

#### Defining Health Behavior Management Targets and Health Education Content

The identification of the 4 key health behavior management targets and associated health education content for hypertension (see Table 1) was based on existing literature and previous research findings.

**Table 1:** Health education targets and content.

Health behaviors	Targets	Health education content
Physical activity (exercise)	Recommended exercise levels according to exercise prescription	Relevant knowledge and exercise skills for personalized intelligent exercise prescriptions
Healthy diet	Choose types and quantities of food according to the DASH^a^ principles	Knowledge related to DASH and techniques for assessing simplified DASH grading diet index score
Taking medication	Adherence to prescribed medication	Knowledge of medications
Monitoring of blood pressure	The recommended frequency of home blood pressure monitoring according to clinical guidelines	Knowledge and skills in blood pressure monitoring

^a^DASH: Dietary Approaches to Stop Hypertension.

#### Development of Digital Health Education Materials

**Figure 7:**
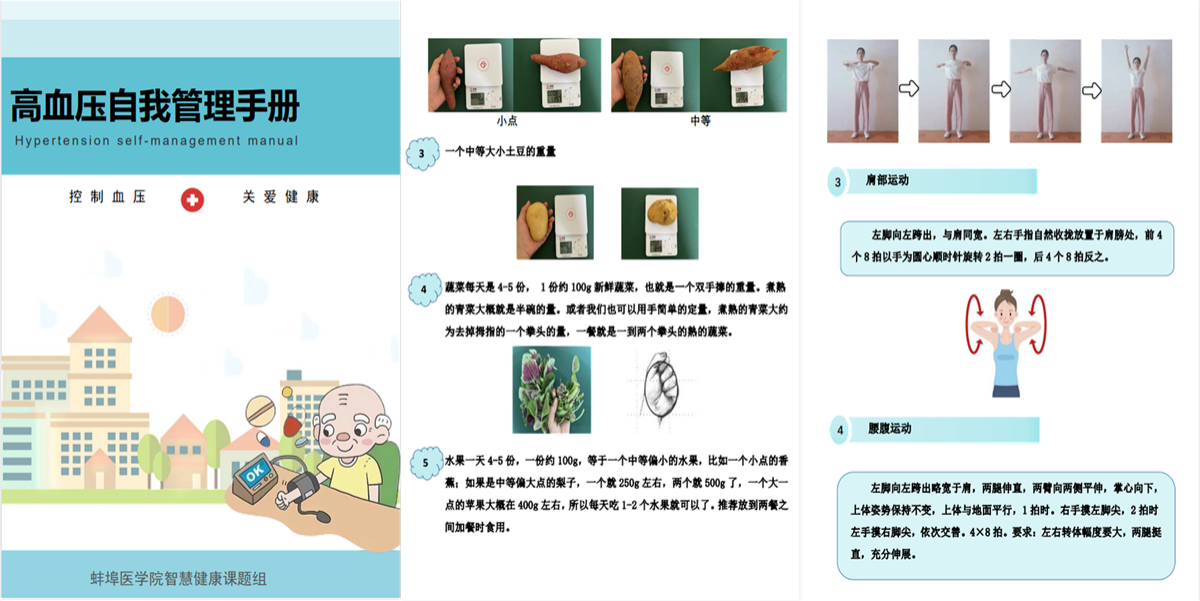
Hypertension self-management manual.

Patient health education needs for the 4 targets were gathered through focus group interviews. Subsequently, evidence-based principles were used to develop textual materials related to exercise, diet, medication taking, and BP monitoring. These materials underwent content validity assessments by experts before being transformed into health education videos. For diet, in addition to creating health education videos based on the DASH guidelines, the research team developed a simplified DASH grading diet index score. The scoring system included items for evaluating daily meals, such as grains, vegetables, fruits, protein sources, cooking oil, and compliance with recommended food types and quantities. Items 1 through 7 represented positive scoring criteria for each recommended food category and quantity, while items 8 through 10 incurred deductions. The aim was to educate patients on recommended dietary behaviors through the acquisition of DASH knowledge and proficiency in using the simplified DASH grading diet index score. For exercise, in addition to providing general knowledge about exercise, the research team augmented the guidance materials (eg, videos on how to perform recommended exercise types) without altering the existing elements of the intelligent exercise prescription. Medication adherence and BP monitoring were primarily addressed through instructional videos that imparted relevant knowledge and skills. All relevant videos were accompanied by textual materials that were ultimately compiled into the “hypertension self-management manual.” See Figure 7 for an overview.

**Development of Digital Intervention Scheme Based on BCW and DMIC**

We used the systematic workflow of the BCW to identify appropriate intervention categories and suitable BCTs. Details of this process have been published in other papers [35]. Based on the identified BCTs, we developed corresponding textual content, which, along with the previously mentioned digital health education materials, collectively form the foundational elements of the DMIC theory. Subsequently, additional elements of the DMIC theory, namely proximal assessment indicators and decision rules, were determined based on literature review and expert opinions.

**Figure 8:**
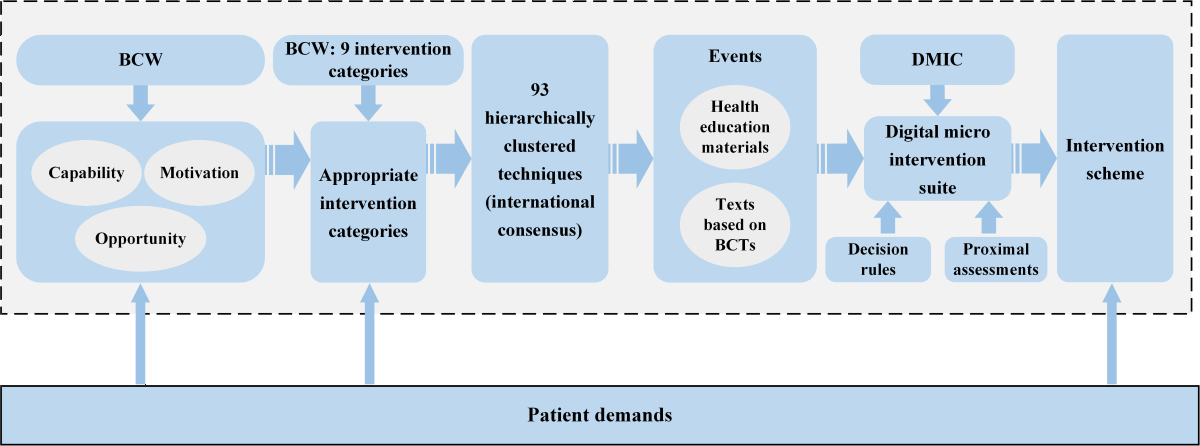
The process of shaping the mobile health (mHealth) intervention scheme for adherence. BCT: behavior change technique; BCW: behavior change wheel; DMIC: digital micro-intervention care.

First, the BCW theory was used to identify barriers related to capability, opportunity, and motivation affecting adherence to health behaviors by patients with hypertension. Intervention categories were chosen to address these barriers, including methods like education, persuasion, and incentivization. Figure 8 illustrates the process of developing the mHealth intervention scheme for improving adherence.

Second, BCTs, such as feedback on behavior, prompts, self-monitoring of behavior, and verbal persuasion about capability, were selected from the BCT taxonomy, which had already been coded and organized by researchers like Michie et al [28]. Furthermore, based on the selected BCT, specific textual content was prepared (eg, BCT: focus on past success; text: “You have successfully quit smoking in the past, and we believe you can also develop a scientific exercise habit! Keep it up!”).

Third, following the DMIC model, we classified the textual content corresponding to BCTs and digital health education materials as “events.” Subsequently, we established decision rules that determined when and in what order interventions for these events should take place. Additionally, we established proximal assessment indicators (eg, knowledge level) for tailoring intervention strategies to individual patients and their corresponding events (eg, continue learning if qualified, relearn if not). Events, proximal assessment, and decision rules were integrated into intervention units organized by chronological stages, including assessing and preparing, committing and planning, and reinforcing behavioral habits. Notably, health education was primarily implemented during the assessing and preparing phase (first week).

Fourth, the intervention scheme was validated by an expert panel through 2 rounds of Delphi surveys. The panel of 15 experts included 6 nutrition experts, 2 clinical cardiologists, 3 clinical nurses and nursing teachers, and 4 exercise experts. The content validity index (CVI) was calculated and assessed using the item-level CVI (I-CVI) and a 4-point scale, respectively. The first round of assessments using the I-CVI ranged from 0.6 to 0.8. After adjusting the content based on the feedback and comments from the experts, the I-CVI reached 1 in the second round.

Finally, all intervention logic and guidance content were compiled into an intervention manual. This manual included daily intervention tasks (eg, questionnaires, text based on BCTs, and the order of videos to be sent), communication guidelines, and personalized guidance strategies (eg, after assessing the extent of the patient's knowledge and skills, the approach to providing personalized guidance can be provided).

### Study Design

#### Trial Design and Setting

**Figure 9:**
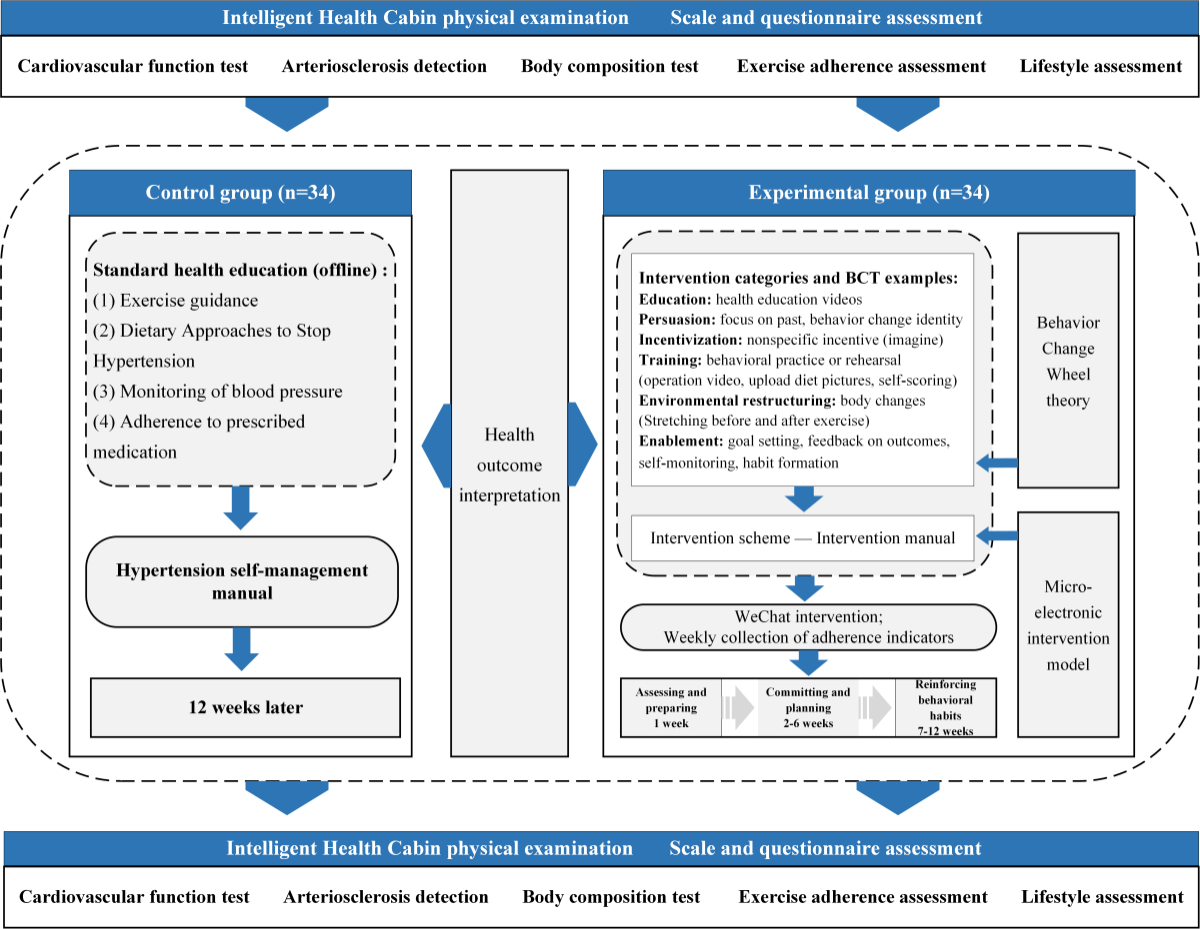
Trial design.

The study protocol was previously published [35]. This was a randomized controlled trial. The experimental group received the health behavioral digital intervention based on an intelligent health promotion system and WeChat for 12 weeks, while the control group received routine health services and was provided with a “Hypertension Self-Management Manual” to guide daily health behaviors (refer to Figure 9). The trial was conducted at 2 community health centers, both located in Anhui Province: Sanxiao Kou Community Health Service Center and Dongfeng Community Health Service Center.

#### Patients

The staff at the community health service centers recruited eligible patients with hypertension within their jurisdiction through phone calls or verbal invitations using a convenience sampling method. The inclusion criteria for participants were as follows: (1) diagnosed with primary hypertension or currently taking antihypertensive medication; (2) aged >60 years; and (3) proficient in using smartphones and the WeChat application. The exclusion criteria were as follows: (1) patients with hypertension undergoing nonpharmacological treatment; (2) those with diabetes, kidney disease, or other conditions requiring special dietary and exercise considerations; (3) individuals participating in or having participated in other health management projects; and (4) those unable to measure BP in a home environment.

#### Sample Size

The sample size for this study was determined based on the effect size. According to a previous meta-analysis comparing the BP-lowering effects of mHealth interventions and other traditional methods used by patients with hypertension, the mHealth experimental group demonstrated a significant reduction in BP, with an effect size of 0.7 [36]. The sample size for this study was calculated using GPower 3.1, assuming α=.05 and β=.2 and accounting for a 20% dropout rate, resulting in a final sample size of 68 participants.

#### Randomization, Allocation, and Blinding

Prior to randomization, a researcher unfamiliar with the experimental design used SPSS (version 23; IBM Corp) to generate 68 random numbers. Subsequently, using the visual binning method, these numbers were divided into 2 groups (experimental group and control group). The paper slips containing the labeled random numbers were placed into sealed envelopes, and another researcher unaware of the experimental design was responsible for assigning participants to either the experimental group or control group. This researcher sequentially opened the envelopes and assigned participants to the experimental group or control group based on the numbers. Additionally, during the data collection phases before and after the experiment, we trained and employed 2 nursing graduate students unfamiliar with the study design. The data collection for the experimental group intervention process was conducted by 5 nursing undergraduates who were also unfamiliar with the experimental design.

#### Outcomes

##### Primary Outcomes

Before and 12 weeks after the intervention, we used a cardiovascular function monitoring device (BX-CFTI-100, Intelligent Machine Institute) to measure participants' BP. Measurement details have been published [13]. Through WeChat, 5 undergraduate students unfamiliar with the experimental design collected adherence indicators from the experimental group participants weekly. These indicators included exercise adherence, calculated as the ratio of actual weekly exercise time meeting the prescribed intensity to the total prescribed weekly exercise time; dietary adherence, assessed using the weekly average score of the simplified DASH grading diet index score (see protocol paper for details [35]); medication adherence, assessed using the weekly average score of the Modified Morisky Scale (Chinese version-MMS-8, Certificate Number: 8538-1877-1559-6025-5310) [37-39]; and BPMA calculated as the ratio of the actual weekly frequency of BP monitoring to the total recommended weekly frequency.

##### Secondary Outcomes

Secondary outcomes were assessed before and after the intervention. Heart rate and subendocardial viability ratio (SEVR) were measured using the cardiovascular function monitor; brachial-ankle pulse wave velocity was measured using an arterial stiffness monitor (BX-AS-100, Intelligent Machine Institute); weight was measured using a body composition analyzer (BX-BCA-100, Intelligent Machine Institute); and lifestyle was assessed through an online questionnaire administered to participants pre- and postintervention. Participants were questioned about health-related behaviors, including the frequency of smoking, drinking, diet, and PA. For smoking, the lifelong smoking quantity of participants was calculated based on the quantity and weekly frequency of cigarettes smoked. For those who had quit smoking, this calculation also included their smoking history before quitting. The alcohol content in one bottle of the most popular alcoholic beverages in Anhui Province is as follows: beer (500 mL, 3.2% alcohol): 17.5 g; white liquor (450 mL, 42% alcohol): 210 g; and wine (750 mL, 13.5%-14% alcohol): 97.5 g. Daily alcohol consumption was calculated using these values. Missing values for smoking and drinking data were set to zero. Participants were asked about the types, duration (in minutes), and frequency (per week) of PA they engaged in. PA time was determined in minutes per metabolic equivalent (MET) per day (min/MET/day) based on activity codes and MET intensities in the “Compendium of Physical Activities.” Weekly exercise time (MET-min/week) and weekly PA time (MET-min/week) were calculated, with missing values set to the median [40].

#### Statistical Analysis

Data analysis was carried out using SPSS, with independent sample *t* tests and chi-square tests used to evaluate the significance of differences between the 2 groups. Paired *t* tests and McNemar tests were used to compare differences within the same group before and after the 3-month intervention. A *P* value <.05 was considered statistically significant.

### Ethical Considerations

The study was approved by the Ethics Committee of Bengbu Medical College in June 2022 under approval number 2022-103, and the study began after informed consent was obtained from patients with hypertension and all participants allowed their data to be used anonymously. An independent data manager conducted weekly checks on the database to ensure its integrity and security. The implementation of data lockup aims to prevent any postmodification. All exported data must undergo anonymization by the data manager before statistical analysis can be conducted to safeguard the participants' information. Each participant who underwent the intervention and data collection at the community health center was given a gift of daily necessities valued at approximately US $6.87.

## Results

### Participants

Between September 5, 2022, and September 19, 2022, a total of 68 patients with hypertension were recruited through phone or verbal invitations from the 2 community health centers in Anhui Province. Participants were re-invited to the community health service center for health data and information collection between December 20, 2022, and January 5, 2023. A total of 54 participants (30 women and 24 men; mean age 67.24, SD 4.19 years) were included in the final analysis: 23 in the experimental group and 31 in the control group. Exclusions were due to various reasons, including hospitalization for illness (2 individuals), inability to complete the postintervention assessment (3 individuals), health conditions deteriorating to the point of hindering PA (4 individuals), voluntary withdrawal from the study (4 individuals), and a change in antihypertensive medication (1 individual). Other patients did not change their hypertension medication during the intervention.

### Baseline Data

No statistically significant differences in health outcomes (eg, SBP, SEVR), adherence indicators (eg, exercise time, PA time, medication adherence), and learning performance were observed between the experimental group and control group at baseline.

### Intervention Effect

Table 2 displays the baseline and posttest results for both groups regarding health outcomes, adherence indicators, and learning performance. Significant changes were observed in SBP (–7.36 mm Hg, *P*=.05), SEVR (0.16, *P*=.01), exercise time (856.35 MET-min/week, *P*=.03), medication adherence (0.56, *P*=.02), BP monitoring frequency (*P*=.046), and learning performance (3.23, *P*<.001) in the intergroup comparison after 12 weeks. The PA time increased for the experimental group in the before-and-after comparisons (*P*=.045). Both groups experienced a reduction in weight after the intervention (experimental: 1.2 kg, *P*=.002; control: 1.11 kg, *P*=.009). Furthermore, the Cohen *d* values reflecting effect size were greater than 0.5 for all variables except PA time, indicating at least an intermediate effect size. Among these variables, the health outcomes of SEVR, recommended diet types (eg, meeting recommendations occasionally, sometimes, often), recommended diet quantities (eg, meeting recommendations occasionally, sometimes, often), BP monitoring frequency (eg, measure daily, measure 1-3 times a week, measure whenever remember), and learning performance had Cohen *d* values >0.8, suggesting a large effect size.

**Table 2:** Effects of the health behavioral digital intervention for hypertensive patients (HBDIHP) effects (N=54).

Measure		Control group				Experimental group				Cohen *d*	*P* value^a^
		Baseline	12 weeks	Cohen *d*	*P* value^a^	Baseline	12 weeks	Cohen *d*	*P* value^a^		
**Health outcomes, mean (SD)**											
	SBP^b^ (mm Hg)	136.94 (18.44)	133.10 (15.02)	0.549	.10	135.43 (17.48)	125.74 (14.76)	1.457	.002	0.636	.05
	DBP^c^ (mm Hg)	76.03 (9.20)	75.58 (6.94)	0.129	.73	78.39 (8.81)	75.96 (6.38)	0.286	.19	0.516	.84
	Heart rate (bpm)	74.58 (10.58)	74.58 (9.45)	0.149	≥.99	77.09 (9.18)	73.91 (9.84)	0.548	.08	0.52	.35
	SEVR^d^	1.11 (0.26)	1.03 (0.15)	0.487	.25	1.12 (0.21)	1.19 (0.25)	0.736	.13	0.806	.01
	baPWV^e^ (m/s)	16.87 (2.48)	16.67 (2.22)	0.21	.64	16.23 (1.82)	16.51 (1.64)	0.4	.51	0.523	.78
	Weight (kg)	69.54 (9.93)	68.34 (9.52)	0.308	.009	71.48 (9.92)	70.37 (9.47)	0.721	.002	0.56	.45
**Adherence indicators, mean (SD)**											
	Exercise time (MET^f^-min/week)	1263.3 (775.50)	1823.74 (1208.06)	1.143	.009	1578.78 (709.95)	2680.09 (1604.09)	1.554	<.001	0.616	.03
	Physical activity time (MET-min/week)	2462.09 (1405.61)	2861.03 (1484.81)	0.688	.15	3166.82 (2277.17)	3742.13 (2138.65)	0.649	.045	0.492	.09
	Medication adherence score	6.99 (1.02)	7.09 (1.00)	0.247	.46	6.77 (1.22)	7.65 (0.49)	1.584	.001	0.68	.02
**Recommended diet types, n (%)**										1.318	<.001
	Occasionally	—^g^	21 (68)	—	—	—	3 (13)	—	—		
	Sometimes	—	8 (26)	—	—	—	6 (26)	—	—		
	Often	—	2 (7)	—	—	—	14 (61)	—	—		
**Recommended diet quantity, n (%)**										1.18	<.001
	Occasionally	—	21 (68)	—	—	—	3 (13)	—	—		
	Sometimes	—	5 (16)	—	—	—	5 (22)	—	—		
	Often	—	5 (16)	—	—	—	15 (65)	—	—		
**Blood pressure monitoring frequency, n (%)**				1.298	<.001			0.654	.02	1.318	.046
	Measure daily	7 (23)	10 (32)			0	12 (52)				
	Measure 1-3 times a week	16 (52)	14 (45)			18 (78)	9 (39)				
	Measure whenever remember	8 (26)	7 (23)			5 (22)	2 (9)				
Learning performance, mean (SD)		9.32 (1.79)	9.55 (1.77)	0.323	.15	9.91 (1.51)	12.78 (2.04)	1.617	<.001	0.887	<.001

^a^12-week intergroup *P* value.

^b^SBP: systolic blood pressure.

^c^DBP: diastolic blood pressure.

^d^SEVR: subendocardial viability ratio.

^e^baPWV: brachial-ankle pulse wave velocity.

^f^MET: metabolic equivalent.

^g^Not applicable.

### Changes in Weekly Adherence Indicators in the Experimental Group

**Figure 10:**
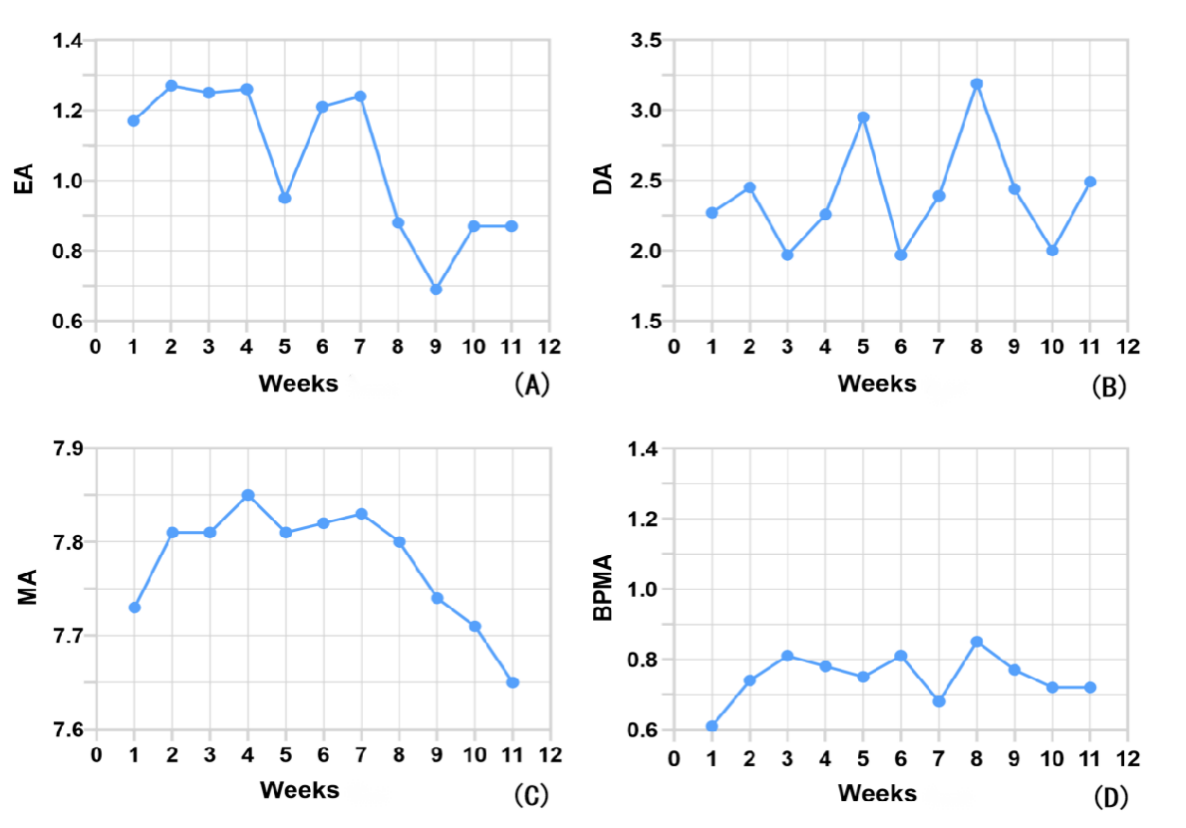
Average adherence indicators by week in the intervention: (A) exercise adherence (EA), (B) dietary adherence (DA), (C) medication adherence (MA), (D) blood pressure monitoring adherence (BPMA).

The experimental group collected weekly average adherence indicators from the first week after the assessing and preparing phase (the second week of the project) through the eleventh week (the twelfth week of the project), as depicted in the curve shown in Figure 10. From weeks 1 to 4, the exercise adherence curve indicated that this phase was when the intervention participants were most actively engaged in PA. They exceeded the prescribed exercise volume (>1). By week 5, the participants showed lower exercise adherence, followed by 2 weeks of rebound. Subsequently, exercise adherence sharply declined, reaching a stable state in weeks 10 and 11. The dietary adherence curve indicators exhibited the most noticeable fluctuations, reaching peaks in weeks 4 and 8 and valleys in weeks 6 and 10. Medication adherence gradually increased from week 1 to week 4, experienced fluctuations, and then steadily declined starting at week 7. Among the 4 indicators, the BPMA curve had smaller fluctuations. It steadily increased from week 1 to week 3, reached a low point in week 7, and remained relatively stable at 0.72 thereafter.

## Discussion

### Health Outcomes

We observed varying degrees of positive impacts on health outcomes for patients with hypertension through this health behavioral digital intervention. The intervention demonstrated a significant effect on SBP control. It may also be effective at improving SEVR (with statistical differences observed between the 2 groups after the intervention). However, there is insufficient evidence to conclude that a WeChat-based digital intervention is more effective than a conventional intervention for weight improvement. Based on the current results, both interventions appear to have a positive effect on weight reduction. No statistically significant differences were found in other health outcome indicators.

The improvements in BP after our program’s intervention were similar to previously published findings. Using methods such as text messaging, electronic reminders, and sharing health education links, both SBP and DBP can be reduced [20-22]. However, our project only demonstrated a significant reduction in SBP and not DBP, consistent with the results found by some scholars [41-43]. The reduction in BP may have resulted from the intervention measures we designed based on BCW and the DMIC model, which enhanced patients’ knowledge and adherence to healthy behaviors. Several behaviors interacted dynamically and influenced each other, such as increased disease knowledge leading to regular BP monitoring at home [8]. Regular attention to BP can assist individuals with better managing their condition [44], ultimately enhancing BP control. Both the DASH diet and exercise, either alone or in combination, can relax the smooth muscle of blood vessel walls to some extent, promoting blood circulation and consequently lowering BP [45,46]. Older adults with decreased vascular elasticity may have a tendency for elevated SBP and decreased DBP (as seen in our study with high mean SBP and normal mean DBP) [47]. They may be more sensitive to reductions in SBP. Another possible reason for the reduction in SBP, but not DBP, could be attributed to variations in study populations, interventions, age groups, and medication usage. Regardless, the results of this study were generally consistent with the antihypertensive effects reported in the guidelines [48]. Additionally, the exercise prescription aligned with the guideline recommendations of engaging in at least 30 minutes of moderate-intensity dynamic aerobic exercise per week (such as walking, jogging, cycling, or swimming) and a minimum of 2 to 3 days of resistance training per week. Simultaneously, the formulation of personalized behavior change strategies has further increased patient adherence to the exercise prescription.

SEVR, a reliable indicator of myocardial oxygen supply and demand [49], may be more closely associated with exercise. Our previous study demonstrated an improvement in this indicator after an exercise intervention [13], and other researchers have also observed significant enhancements in SEVR across different age groups (18-80 years) following exercise interventions [50]. Regarding weight, there was a reduction observed in both groups of patients with hypertension when comparing pre- and postintervention data. This reduction aligns with the results of our previous 1-arm before-and-after study [13]. However, the WeChat-based intervention did not demonstrate superiority, possibly due to the limited sample size with this experiment. There were no statistically significant differences observed in heart rate and brachial-ankle pulse wave velocity. This finding contradicted our 1-year study results, which may be attributed to the small sample size and the relatively short duration of the study. Some scholars pointed out that exercise training to improve atherosclerosis requires higher exercise intensity and longer duration, while the participants in this study were older adults with chronic diseases and the exercise prescriptions mostly provided lower exercise intensity [51].

### Adherence Indicators

This program enhanced the health knowledge of community-based patients with hypertension and fostered compliance with medication, BP monitoring, exercise, and dietary guidelines, which was consistent with the findings in some previous literature [20-22]. The improvement in knowledge highlighted the efficacy of educating patients with hypertension, serving as the cornerstone of healthy behavior change.

The improvement in medication adherence in our research supports the findings of other research [52-54]. This study indicated that the Morisky scores before and after the intervention (6.77-7.65) were within the moderate range, with slightly better outcomes compared with the results reported by Morawski et al [55] (same assessment tool: 12 weeks, 6-6.3). Medication education and adherence reminders based on video, text messages, and other mobile app functions were some of the most common interventions for medication adherence in cardiovascular diseases [56]. These were key BCTs in our project. Foreman et al [57] suggested that medication text reminders reinforcing medication adherence can lead to higher oral medication adherence among patients with hypertension. Information reminders also help patients maintain higher compliance over time. However, long-term, high-frequency reminders may result in response fatigue in patients [58]. This may explain why our research showed that medication adherence was highest during weeks 2 to 6 (commitment and planning stage) but significantly declined after week 7 (behavioral habit consolidation stage). This highlights the need for researchers to adopt additional behavior change strategies to address patient fatigue during this phase. The effectiveness of electronic reminders and health education in improving patient compliance with BP monitoring has also been confirmed [53]. Based on our research findings, the curve for BPMA showed the least fluctuation. This suggests that BPMA is relatively stable for patients. It is crucial to emphasize the importance of BP monitoring to patients and establish corresponding health behaviors, particularly during the initial phase of the intervention. Additionally, monitoring BP is often the first health behavior action adopted by patients, and it plays a pivotal role in the transition to other health behaviors [44]. Therefore, it should receive special attention during the initial stages.

Most studies have consistently demonstrated the impact of various exercise types and intensities on BP improvement. Research involving mHealth or telehealth interventions through smartphones for patients with coronary heart disease or hypertension has also reported their positive effects on exercise compliance [22,59], which were consistent with our results. Our research revealed a significant rise and reduction in the exercise prescription adherence curve. This indicator was highest during weeks 2 to 6, but it significantly declined starting from week 7. This suggests that emphasis should be placed on maintaining exercise habits. The effectiveness of DASH in improving BP is unquestionable. However, the results of a systematic review indicated only weak evidence supporting the use of smartphone apps to enhance DASH dietary adherence and reduce BP [60]. From our study's perspective, there was significant fluctuation in DASH dietary adherence. This variability may be attributed to the complexity of dietary management compared with other health behaviors, making it difficult to implement and provide feedback [61]. This poses a greater challenge for researchers in designing interventions to improve dietary compliance among patients with hypertension.

In this study, considering the decline in participant adherence indicators after a certain period of intervention, effective strategies may help alleviate participant adherence fatigue, thereby sustaining and enhancing patient engagement. For exercise adherence, research indicates that the effectiveness of interventions is not necessarily correlated with longer intervention periods or higher frequencies. Therefore, tailoring interventions to individual preferences, using different proven therapeutic intervention types for specific target populations, maintaining intervention frequencies above once per week, and ensuring a moderate planned duration may be crucial factors in promoting intervention adherence [62]. Additionally, involving professionals from different disciplines (such as psychologists, doctors, and nurses), having professionals supervise the implementation of intervention plans, and actively engaging in social interactions with staff and other participants have proven effective in enhancing participant adherence and increasing engagement [62]. Regular home visits [63], actively implementing strategies to increase participant self-efficacy [64], and incorporating gamification elements into interventions [65] are also effective strategies to address participant compliance fatigue. During the intervention implementation process, dynamically assessing and understanding the drivers and barriers of adherence based on the participant's stage and providing personalized decision support and motivation can effectively enhance participant adherence and engagement [66,67].

Notably, there is a lack of research on behavior change interventions for hypertension based on the BCW and DMIC. Therefore, a more intricate experimental design and thorough investigation are required to understand the precise mechanisms underlying the effectiveness of this project, including which components and specific BCTs are effective.

### Limitations

This study has several limitations. First, we were unable to implement blinding for the personnel involved in the hypertensive health behavior interventions. To mitigate this, we established standardized intervention procedures, provided intervention strategies tailored to different patient types, edited intervention guidance language based on BCTs, incorporated the aforementioned content into the intervention manual, and conducted training for all intervention personnel. Second, the generalizability of our trial's results may be limited for populations of patients with hypertension residing elsewhere, as they may possess sociodemographic and comorbidity characteristics distinct from those of our study participants. Third, some measurement indicators relied on patient self-report, which could potentially affect the credibility of the results. Fourth, considering the workload, this study did not assess changes in compliance indicators in the control group nor did it collect more comprehensive dietary-related information. This requires correction in future experiments of app-based hypertension health behavior interventions.

### Conclusions

The observations suggest that our program may have improved specific health outcomes and adherence to health behaviors in older adults with hypertension. In terms of health outcomes, participants observed significant improvements in SBP, SEVR, and weight. Moreover, there were noteworthy changes in adherence indicators, such as exercise duration, medication adherence, PA duration, frequency of BP monitoring, and learning performance. However, due to our small sample size and short intervention duration, a larger sample size and longer randomized controlled trial are needed to validate the intervention's effects, explore its mechanisms, and identify the specific design elements that are effective. Additionally, among the 4 adherence behaviors, dietary adherence is the most susceptible to external influences, and more BCTs targeting dietary adherence should be considered in intervention design.

## Appendix

Multimedia Appendix 1 Consort E-health V1.6.

## Abbreviations

BCT: behavior change technique
BCW: behavior change wheel
BP: blood pressure
BPMA: blood pressure monitoring adherence
COM-B: Capability, Opportunity, Motivation-Behavior
CVI: content validity index
DA: dietary adherence
DASH: Dietary Approaches to Stop Hypertension
DBP: diastolic blood pressure
DMIC: digital micro-intervention care
FITT-VP: frequency, intensity, type, time, volume, and progression
HBDIHP: health behavioral digital intervention for hypertensive patients
I-CVI: item-level content validity index
MET: metabolic equivalent
mHealth: mobile health
PA: physical activity
SBP: systolic blood pressure
SEVR: subendocardial viability ratio
