# Supplementary material for: Development of a Health Behavioral Digital Intervention for Patients With Hypertension Based on an Intelligent Health Promotion System and WeChat: Randomized Controlled Trial
Source: JMIR Mhealth Uhealth. 2024 Apr 5;12:e53006. doi: 10.2196/53006 (PMC11031705; doi:10.2196/53006)
Supplement: Multimedia Appendix 1 [file mhealth_v12i1e53006_app1.pdf]

CONSORT-EHEALTH (V 1.6.1) - Submission/Publication Form

The CONSORT-EHEALTH checklist is intended for authors of randomized trials evaluating web-based and Internet-based applications/interventions, including mobile interventions, electronic games (incl multiplayer games), social media, certain telehealth applications, and other interactive and/or networked electronic applications. Some of the items (e.g. all subitems under item 5 - description of the intervention) may also be applicable for other study designs.

The goal of the CONSORT EHEALTH checklist and guideline is to be
a) a guide for reporting for authors of RCTs,
b) to form a basis for appraisal of an ehealth trial (in terms of validity)

CONSORT-EHEALTH items/subitems are MANDATORY reporting items for studies published in the Journal of Medical Internet Research and other journals / scientific societies endorsing the checklist.

Items numbered 1., 2., 3., 4a., 4b etc are original CONSORT or CONSORT-NPT (non-pharmacologic treatment) items.
Items with Roman numerals (i., ii, iii, iv etc.) are CONSORT-EHEALTH extensions/clarifications.

As the CONSORT-EHEALTH checklist is still considered in a formative stage, we would ask that you also RATE ON A SCALE OF 1-5 how important/useful you feel each item is FOR THE PURPOSE OF THE CHECKLIST and reporting guideline (optional).

Mandatory reporting items are marked with a red *.
In the textboxes, either copy & paste the relevant sections from your manuscript into this form - please include any quotes from your manuscript in QUOTATION MARKS,
or answer directly by providing additional information not in the manuscript, or elaborating on why the item was not relevant for this study.

YOUR ANSWERS WILL BE PUBLISHED AS A SUPPLEMENTARY FILE TO YOUR PUBLICATION IN JMIR AND ARE CONSIDERED PART OF YOUR PUBLICATION (IF ACCEPTED).
Please fill in these questions diligently. Information will not be copyedited, so please use proper spelling and grammar, use correct capitalization, and avoid abbreviations.

DO NOT FORGET TO SAVE AS PDF _AND_ CLICK THE SUBMIT BUTTON SO YOUR ANSWERS ARE IN OUR DATABASE !!!

Citation Suggestion (if you append the pdf as Appendix we suggest to cite this paper in the caption):
Eysenbach G, CONSORT-EHEALTH Group
[illegible]

您的回复过长。请尝试让您的部分回答更简短。

[illegible]

URL: http://www.jmir.org/2011/4/e126/
doi: 10.2196/jmir.1923
PMID: 22209829

3542287675@qq.com 切换账号

未共享的内容

草稿已保存

* 表示必填

Your name *

First Last

Ting Sun

Primary Affiliation (short), City, Country *

University of Toronto, Toronto, Canada

Bengbu Medical University, Bengbu, Anhui, Chi

Your e-mail address *

abc@gmail.com

cangwuge21@163.com

Title of your manuscript *

Provide the (draft) title of your manuscript.

Health Behavioral Digital Intervention for Hypertensive Patients Based on Intelligent Health Promotion System and WeChat: Development and Evaluation

您的回复过长。请尝试让您的部分回答更简短。

Name of your App/Software/Intervention *

If there is a short and a long/alternate name, write the short name first and add the long name in brackets.

Intelligent Health Promotion System and WeCh

Evaluated Version (if any)

e.g. "V1", "Release 2017-03-01", "Version 2.0.27913"

您的回答

Language(s) *

What language is the intervention/app in? If multiple languages are available, separate by comma (e.g. "English, French")

Chinese

URL of your Intervention Website or App

e.g. a direct link to the mobile app on app in appstore (itunes, Google Play), or URL of the website. If the intervention is a DVD or hardware, you can also link to an Amazon page.

您的回答

URL of an image/screenshot (optional)

您的回答

您的回复过长。请尝试让您的部分回答更简短。

Accessibility *

Can an enduser access the intervention presently?

- (x) access is free and open
- ( ) access only for special usergroups, not open
- ( ) access is open to everyone, but requires payment/subscription/in-app purchases
- ( ) app/intervention no longer accessible
- ( ) 其他:

Primary Medical Indication/Disease/Condition *

e.g. "Stress", "Diabetes", or define the target group in brackets after the condition, e.g. "Autism (Parents of children with)", "Alzheimers (Informal Caregivers of)"

Hypertensive Patients

Primary Outcomes measured in trial *

comma-separated list of primary outcomes reported in the trial

blood pressure； exercise adherence； dietary

Secondary/other outcomes

Are there any other outcomes the intervention is expected to affect?

您的回答

您的回复过长。请尝试让您的部分回答更简短。

Recommended "Dose" *

What do the instructions for users say on how often the app should be used?

- ○ Approximately Daily
- ○ Approximately Weekly
- ○ Approximately Monthly
- ○ Approximately Yearly
- ◉ "as needed"
- ○ 其他:

Approx. Percentage of Users (starters) still using the app as recommended after 3 months *

- ○ unknown / not evaluated
- ○ 0-10%
- ○ 11-20%
- ○ 21-30%
- ○ 31-40%
- ○ 41-50%
- ○ 51-60%
- ○ 61-70%
- ◉ 71%-80%
- ○ 81-90%
- ○ 91-100%
- ○ 其他:

您的回复过长。请尝试让您的部分回答更简短。

Overall, was the app/intervention effective? *

- ◯ yes: all primary outcomes were significantly better in intervention group vs control
- ◉ partly: SOME primary outcomes were significantly better in intervention group vs control
- ◯ no statistically significant difference between control and intervention
- ◯ potentially harmful: control was significantly better than intervention in one or more outcomes
- ◯ inconclusive: more research is needed
- ◯ 其他:

Article Preparation Status/Stage *

At which stage in your article preparation are you currently (at the time you fill in this form)

- ◯ not submitted yet - in early draft status
- ◯ not submitted yet - in late draft status, just before submission
- ◯ submitted to a journal but not reviewed yet
- ◉ submitted to a journal and after receiving initial reviewer comments
- ◯ submitted to a journal and accepted, but not published yet
- ◯ published
- ◯ 其他:

您的回复过长。请尝试让您的部分回答更简短。

Journal *

If you already know where you will submit this paper (or if it is already submitted), please provide the journal name (if it is not JMIR, provide the journal name under "other")

- ○ not submitted yet / unclear where I will submit this
- ◉ Journal of Medical Internet Research (JMIR)
- ○ JMIR mHealth and UHealth
- ○ JMIR Serious Games
- ○ JMIR Mental Health
- ○ JMIR Public Health
- ○ JMIR Formative Research
- ○ Other JMIR sister journal
- ○ 其他:

Is this a full powered effectiveness trial or a pilot/feasibility trial? *

- ○ Pilot/feasibility
- ◉ Fully powered

Manuscript tracking number *

If this is a JMIR submission, please provide the manuscript tracking number under "other" (The ms tracking number can be found in the submission acknowledgement email, or when you login as author in JMIR. If the paper is already published in JMIR, then the ms tracking number is the four-digit number at the end of the DOI, to be found at the bottom of each published article in JMIR)

- ○ no ms number (yet) / not (yet) submitted to / published in JMIR
- ◉ 其他: ms #53006

您的回复过长。请尝试让您的部分回答更简短。

TITLE AND ABSTRACT

1a) TITLE: Identification as a randomized trial in the title

1a) Does your paper address CONSORT item 1a? *

I.e does the title contain the phrase "Randomized Controlled Trial"? (if not, explain the reason under "other")

- ○ yes
- ◉ 其他: None

1a-i) Identify the mode of delivery in the title

Identify the mode of delivery. Preferably use “web-based” and/or “mobile” and/or “electronic game” in the title. Avoid ambiguous terms like “online”, “virtual”, “interactive”. Use “Internet-based” only if Intervention includes non-web-based Internet components (e.g. email), use “computer-based” or “electronic” only if offline products are used. Use “virtual” only in the context of “virtual reality” (3-D worlds). Use “online” only in the context of “online support groups”. Complement or substitute product names with broader terms for the class of products (such as “mobile” or “smart phone” instead of “iphone”), especially if the application runs on different platforms.

	1	2	3	4	5	
subitem not at all important	○	○	◉	○	○	essential

清除所选内容

Does your paper address subitem 1a-i? *

Copy and paste relevant sections from manuscript title (include quotes in quotation marks "like this" to indicate direct quotes from your manuscript), or elaborate on this item by providing additional information not in the ms, or briefly explain why the item is not applicable/relevant for your study

Like this: "Based on Intelligent Health Promotion System and WeChat"

您的回复过长。请尝试让您的部分回答更简短。

1a-ii) Non-web-based components or important co-interventions in title

Mention non-web-based components or important co-interventions in title, if any (e.g., “with telephone support”).

	1	2	3	4	5	
subitem not at all important	○	○	○	●	○	essential

清除所选内容

Does your paper address subitem 1a-ii?

Copy and paste relevant sections from manuscript title (include quotes in quotation marks "like this" to indicate direct quotes from your manuscript), or elaborate on this item by providing additional information not in the ms, or briefly explain why the item is not applicable/relevant for your study

Like this:"Based on Intelligent Health Promotion System and WeChat"

1a-iii) Primary condition or target group in the title

Mention primary condition or target group in the title, if any (e.g., “for children with Type I Diabetes”) Example: A Web-based and Mobile Intervention with Telephone Support for Children with Type I Diabetes: Randomized Controlled Trial

	1	2	3	4	5	
subitem not at all important	○	○	○	●	○	essential

清除所选内容

Does your paper address subitem 1a-iii? *

Copy and paste relevant sections from manuscript title (include quotes in quotation marks "like this" to indicate direct quotes from your manuscript), or elaborate on this item by providing additional information not in the ms, or briefly explain why the item is not applicable/relevant for your study

Like this:"for Hypertensive Patients"

您的回复过长。请尝试让您的部分回答更简短。

1b) ABSTRACT: Structured summary of trial design, methods, results, and conclusions

NPT extension: Description of experimental treatment, comparator, care providers, centers, and blinding status.

1b-i) Key features/functionalities/components of the intervention and comparator in the METHODS section of the ABSTRACT

Mention key features/functionalities/components of the intervention and comparator in the abstract. If possible, also mention theories and principles used for designing the site. Keep in mind the needs of systematic reviewers and indexers by including important synonyms. (Note: Only report in the abstract what the main paper is reporting. If this information is missing from the main body of text, consider adding it)

	1	2	3	4	5	
subitem not at all important	○	○	○	●	○	essential

清除所选内容

您的回复过长。请尝试让您的部分回答更简短。

Does your paper address subitem 1b-i? *

Copy and paste relevant sections from the manuscript abstract (include quotes in quotation marks "like this" to indicate direct quotes from your manuscript), or elaborate on this item by providing additional information not in the ms, or briefly explain why the item is not applicable/relevant for your study

Like this:"Background: The effectiveness of timely medication, physical activity (PA), a healthy diet, and blood pressure (BP) monitoring in promoting health outcomes and behavioral changes among hypertensive patients, is supported by a substantial amount of literature, with "adherence" playing a pivotal role. Nevertheless, there is a lack of consistent evidence regarding whether digital interventions can improve adherence to healthy behaviors among hypertensive individuals.
Objectives: The aim was to develop a health behavioral digital intervention for hypertensive patients (HBDIHP) based on an intelligent health promotion system and WeChat following the Behaviour Change Wheel (BCW) theory and digital micro-intervention care (DMIC) model and assess its efficacy in controlling BP and improving healthy behavior adherence.
Methods: A two-arm, randomized trial design was employed. Sixty-eight hypertensive patients aged over 60 were randomly assigned in a 1:1 ratio to either the control or experimental group. The digital intervention was established through the following steps: 1. Developing digital health education materials focused on adherence to exercise prescriptions, Dietary Approaches to Stop Hypertension (DASH), prescribed medication, and monitoring of BP. 2. Utilizing the BCW theory to select behavior change techniques (BCTs) 3. Constructing the intervention's logic following the guidelines of the DMIC model. 4. Creating an intervention manual including the elements above. Prior to the experiment, participants underwent physical examinations at the community health service center's intelligent health cabin and received intelligent personalized health recommendations. The experimental group underwent a 12-week behavior intervention via WeChat, while the control group received routine health education and a self-management manual. The primary outcomes include BP and adherence indicators. Data analysis was performed using SPSS, with independent sample t-tests, chi-square tests, paired t-tests, and McNemar's tests employed. A P-value less than .05 was considered statistically significant.
Results: The final analysis included 54 participants with a mean age of 67.24±4.19 years, comprising 23 individuals in the experimental group and 31 in the control group. Improvements were observed in systolic blood pressure (SBP:-7.36 mmHg), exercise time(+856.35 MET-min/week), medication adherence (MA:+0.56), BP monitoring frequency (P<.05), diet types/amounts(P<.05), subendocardial viability ratio (SEVR:+0.16), and learning performance (+ 3.23) among experimental group participants (P<.05). Both groups showed a reduction in weight (1.2 and 1.11 kg) after intervention (P<.05). However, there were no statistically significant changes in other health outcomes (P>.05). The adherence curve for exercise and medication displayed a notable decline compared to the first week after health education. The dietary adherence (DA) curve exhibited substantial fluctuations, whereas blood pressure monitoring adherence (BPMA) showed the smallest degree of variation.
Conclusions: The observations suggest our program may have enhanced specific health outcomes and adherence to health behaviors in elderly hypertensive patients. However, it is necessary to conduct longer-term and larger-scale trial to validate the effectiveness."

您的回复过长。请尝试让您的部分回答更简短。

1b-ii) Level of human involvement in the METHODS section of the ABSTRACT

Clarify the level of human involvement in the abstract, e.g., use phrases like “fully automated” vs. “therapist/nurse/care provider/physician-assisted” (mention number and expertise of providers involved, if any). (Note: Only report in the abstract what the main paper is reporting. If this information is missing from the main body of text, consider adding it)

	1	2	3	4	5	
subitem not at all important	○	○	○	○	○	essential

Does your paper address subitem 1b-ii?

Copy and paste relevant sections from the manuscript abstract (include quotes in quotation marks "like this" to indicate direct quotes from your manuscript), or elaborate on this item by providing additional information not in the ms, or briefly explain why the item is not applicable/relevant for your study

您的回答

1b-iii) Open vs. closed, web-based (self-assessment) vs. face-to-face assessments in the METHODS section of the ABSTRACT

Mention how participants were recruited (online vs. offline), e.g., from an open access website or from a clinic or a closed online user group (closed usergroup trial), and clarify if this was a purely web-based trial, or there were face-to-face components (as part of the intervention or for assessment). Clearly say if outcomes were self-assessed through questionnaires (as common in web-based trials). Note: In traditional offline trials, an open trial (open-label trial) is a type of clinical trial in which both the researchers and participants know which treatment is being administered. To avoid confusion, use “blinded” or “unblinded” to indicated the level of blinding instead of “open”, as “open” in web-based trials usually refers to “open access” (i.e. participants can self-enrol). (Note: Only report in the abstract what the main paper is reporting. If this information is missing from the main body of text, consider adding it)

	1	2	3	4	5	
subitem not at all important	○	○	○	○	○	essential

您的回复过长。请尝试让您的部分回答更简短。

Does your paper address subitem 1b-iii?

Copy and paste relevant sections from the manuscript abstract (include quotes in quotation marks "like this" to indicate direct quotes from your manuscript), or elaborate on this item by providing additional information not in the ms, or briefly explain why the item is not applicable/relevant for your study

您的回答

1b-iv) RESULTS section in abstract must contain use data

Report number of participants enrolled/assessed in each group, the use/uptake of the intervention (e.g., attrition/adherence metrics, use over time, number of logins etc.), in addition to primary/secondary outcomes. (Note: Only report in the abstract what the main paper is reporting. If this information is missing from the main body of text, consider adding it)

	1	2	3	4	5	
subitem not at all important	○	○	○	○	○	essential

Does your paper address subitem 1b-iv?

Copy and paste relevant sections from the manuscript abstract (include quotes in quotation marks "like this" to indicate direct quotes from your manuscript), or elaborate on this item by providing additional information not in the ms, or briefly explain why the item is not applicable/relevant for your study

您的回答

您的回复过长。请尝试让您的部分回答更简短。

1b-v) CONCLUSIONS/DISCUSSION in abstract for negative trials

Conclusions/Discussions in abstract for negative trials: Discuss the primary outcome - if the trial is negative (primary outcome not changed), and the intervention was not used, discuss whether negative results are attributable to lack of uptake and discuss reasons. (Note: Only report in the abstract what the main paper is reporting. If this information is missing from the main body of text, consider adding it)

	1	2	3	4	5	
subitem not at all important	○	○	○	○	○	essential

Does your paper address subitem 1b-v?

Copy and paste relevant sections from the manuscript abstract (include quotes in quotation marks "like this" to indicate direct quotes from your manuscript), or elaborate on this item by providing additional information not in the ms, or briefly explain why the item is not applicable/relevant for your study

您的回答

INTRODUCTION

2a) In INTRODUCTION: Scientific background and explanation of rationale

2a-i) Problem and the type of system/solution

Describe the problem and the type of system/solution that is object of the study: intended as stand-alone intervention vs. incorporated in broader health care program? Intended for a particular patient population? Goals of the intervention, e.g., being more cost-effective to other interventions, replace or complement other solutions? (Note: Details about the intervention are provided in “Methods” under 5)

	1	2	3	4	5	
subitem not at all important	○	○	○	○	○	essential

您的回复过长。请尝试让您的部分回答更简短。

Does your paper address subitem 2a-i? *

Copy and paste relevant sections from the manuscript (include quotes in quotation marks "like this" to indicate direct quotes from your manuscript), or elaborate on this item by providing additional information not in the ms, or briefly explain why the item is not applicable/relevant for your study

您的回复过长。请尝试让您的部分回答更简短。

Like this:"Hypertension and Health Behavior Interventions
Hypertension, as a high-prevalence chronic disease, has become an important risk factor for many diseases (e.g., stroke, and renal disease) and a major contributor to the global burden of disease[1,2]. About one-third of elderly patients with hypertension failed to achieve their blood pressure (BP) control goals[3]. The reasons for the low rate of hypertension control are related to high-risk lifestyles such as poor dietary habits and low levels of physical activity (PA). Relevant studies have shown that adherence to recommended health behaviors can significantly reduce systolic blood pressure (SBP) by 4.0-5.6 mmHg and diastolic blood pressure (DBP) by 4.1-5.3 mmHg in hypertensive patients[4]. Engaging in a wide range of exercise training can lead to reductions of 4.08-8.24 mmHg in SBP and 2.5-4.0 mmHg in DBP, respectively[5]; Regular BP monitoring behaviors result in reductions of 2.53-4.7 mmHg in SBP and 1.45-2.4 mmHg in DBP, respectively[6,7]; and adherence to Dietary Approaches to Stop Hypertension (DASH) not only reduces SBP and DBP by 5.5 mmHg and 3mmHg respectively but also reduced the chance of developing hypertension by 26%[8,9]. High medication adherence (MA), combined with comprehensive interventions like diet and exercise management, leads to improved BP control[10,11]. It is evident that comprehensive health behavior interventions can achieve effective BP control, with adherence playing a pivotal role.
In recent years, several studies exploring intelligent health promotion systems, incorporating advanced technologies like artificial intelligence, wearable devices, and mobile communication, have consistently shown their efficacy in managing chronic diseases[12–14]. In addition, numerous literature support independent mHealth applications also play a vital role in community-based patient management [10,12,14–16]. WeChat, China's predominant social communication mobile application, serves as one of platforms for mHealth interventions. It boasts a staggering daily user count of up to 902 million and over 1 billion monthly active users spanning all age groups[17]. Several studies have indicated that mHealth-based interventions can enhance health outcomes, quality of life, and self-care among patients with chronic diseases[18,19]. Blood pressure monitoring adherence (BPMA), dietary habits, and self-efficacy behaviors of hypertensive patients has been somewhat improved by these interventions based on mhealth [20–22]. However, other studies have shown no significant change or little improvement in BP, medicine, exercise, and DASH adherence[20,23–25].
Theoretical Framework
The Behavior Change Wheel (BCW), which integrates 19 relevant theoretical frameworks for behaviour change, was first proposed by Michie in 2011[26,27]. As Figure 1 illustrates, it consists of three tiers: the inner tier is the Capability, Opportunity, Motivation-Behaviour (COM-B) model, which is used to identify barriers to intervening in the target behaviour. The second tier comprises nine intervention categories: education, persuasion, motivation, coercion, training, restriction, environmental restructuring, demonstration, and empowerment, intended to tackle identified behavioral obstacles. The outermost tier encompasses seven policy categories (e.g., regulation and legislation) that aid in the implementation of macro-level interventions[27]. This theory consists of three steps: understanding the behavior, identifying intervention options, and identifying content and implementing the options (i.e., behavior change techniques, BCTs). Michie, along with other scholars, developed the “The behavior change technique taxonomy of 93 hierarchically clustered techniques,”which contains 93 BCTs and provides names, definitions, and examples[28]. In the final step, researchers can select the necessary BCTs from this taxonomy list.
[illegible]

您的回复过长。请尝试让您的部分回答更简短。

[illegible]
The effectiveness of this lies

in its ability to aid interveners in the systematic and scientific identification of intervention functions and specific BCTs for behavior change problems. Despite this, the theory does not provide much insight into the components of the intervention. The BCW can be supplemented by the digital micro-intervention care (DMIC) model[25,34].
In 2020, the DMIC model proposed by Amit Baumel and colleagues[34] provided a reference paradigm. This theoretical model promotes shorter, more focused interventions, known as 'micro-interventions'. It can be highly focused on implementation in people's daily lives to help intervention recipients achieve desired short-term goals (the basis for achieving long-term goals). DMIC comprises three core concepts: Events (in-the-moment attempts for change or impact toward the overall target of the intervention), Decision rules (guiding which events are deployed and when), and Proximal assessments (assessing the impact of the event). Events are similar to specific BCTs, while Decision rules deploy events in a meaningful way based on time, user status, or environmental information, allowing interveners to dynamically adjust the content of micro-interventions. Overall outcome assessment, proximal event outcome assessment, and assessment of user participation in the micro-intervention are three types of impact assessment for digital micro-interventions. Based on the results of the overall and proximal assessments and the continuous recording of the user engagement experience (i.e., measuring the quality of attention, engagement, and immersion during the use of the program), it is possible to identify individuals with low levels of engagement and later search for the causes, modify the intervention decision rules, and re-engage the user.This theoretical framework advocates for interventions that aim to achieve specific objectives through in-the-moment intervention elements. These elements may not be directly tied to the attainment of a broader clinical goal [34].Each intervention in every event represents an immediate attempt to modify or influence the overall goal of the intervention. This implies that, in order to achieve a clinical objective, interventions should be broken down into numerous small steps, regulated through proximal assessments, ultimately leading to the attainment of the overall outcome. "

2a-ii) Scientific background, rationale: What is known about the (type of) system

Scientific background, rationale: What is known about the (type of) system that is the object of the study (be sure to discuss the use of similar systems for other conditions/diagnoses, if appropiate), motivation for the study, i.e. what are the reasons for and what is the context for this specific study, from which stakeholder viewpoint is the study performed, potential impact of findings [2]. Briefly justify the choice of the comparator.

	1	2	3	4	5	
subitem not at all important	○	○	○	○	○	essential

您的回复过长。请尝试让您的部分回答更简短。

Does your paper address subitem 2a-ii? *

Copy and paste relevant sections from the manuscript (include quotes in quotation marks "like this" to indicate direct quotes from your manuscript), or elaborate on this item by providing additional information not in the ms, or briefly explain why the item is not applicable/relevant for your study

Like this:"In recent years, several studies exploring intelligent health promotion systems, incorporating advanced technologies like artificial intelligence, wearable devices, and mobile communication, have consistently shown their efficacy in managing chronic diseases[12–14]. In addition, numerous literature support independent mHealth applications also play a vital role in community-based patient management [10,12,14–16]. WeChat, China's predominant social communication mobile application, serves as one of platforms for mHealth interventions. It boasts a staggering daily user count of up to 902 million and over 1 billion monthly active users spanning all age groups[17]. Several studies have indicated that mHealth-based interventions can enhance health outcomes, quality of life, and self-care among patients with chronic diseases[18,19]. Blood pressure monitoring adherence (BPMA), dietary habits, and self-efficacy behaviors of hypertensive patients has been somewhat improved by these interventions based on mhealth [20–22]. However, other studies have shown no significant change or little improvement in BP, medicine, exercise, and DASH adherence[20,23–25]. "; "Scholars have applied this theory to community health promotion, healthcare management, and nursing care, resulting in positive outcomes[29–32]. Additionally, some researchers have extended its application to digital and mobile health interventions[33]. The effectiveness of this lies
in its ability to aid interveners in the systematic and scientific identification of intervention functions and specific BCTs for behavior change problems. Despite this, the
theory does not provide much insight into the components of the intervention. The BCW can be supplemented by the digital micro-intervention care (DMIC) model[25,34]. "

2b) In INTRODUCTION: Specific objectives or hypotheses

Does your paper address CONSORT subitem 2b? *

Copy and paste relevant sections from the manuscript (include quotes in quotation marks "like this" to indicate direct quotes from your manuscript), or elaborate on this item by providing additional information not in the ms, or briefly explain why the item is not applicable/relevant for your study

Like this:"Therefore, our objective was to develop a health behavioral digital intervention for hypertensive patients (HBDIHP) based on BCW and DMIC and assess the effectiveness of this program in two groups after three months of intervention. This program involved exercise, diet, BP monitoring, and MA intervention strategies. Consequently, we aimed to assess the effectiveness of this approach in enhancing outcomes for elderly individuals

您的回复过长。请尝试让您的部分回答更简短。

METHODS

3a) Description of trial design (such as parallel, factorial) including allocation ratio

Does your paper address CONSORT subitem 3a? *

Copy and paste relevant sections from the manuscript (include quotes in quotation marks "like this" to indicate direct quotes from your manuscript), or elaborate on this item by providing additional information not in the ms, or briefly explain why the item is not applicable/relevant for your study

Like this:"The study protocol was previously published[35]. This was a randomized controlled trial. The experimental group received health behavioral digital intervention based on an intelligent health promotion system and WeChat for 12 weeks, while The control group received routine health services and was provided with a "Hypertension Self-Management Manual" to guide daily health behaviors (refer to Figure 9).The trial is conducted at two community health centers: Sanxiao Kou Community Health Service Center and Dongfeng Community Health Service Center, both located in Anhui province. "

3b) Important changes to methods after trial commencement (such as eligibility criteria), with reasons

Does your paper address CONSORT subitem 3b? *

Copy and paste relevant sections from the manuscript (include quotes in quotation marks "like this" to indicate direct quotes from your manuscript), or elaborate on this item by providing additional information not in the ms, or briefly explain why the item is not applicable/relevant for your study

None. The systems and applications used are relatively mature and stable, with no major changes in approach or content.

您的回复过长。请尝试让您的部分回答更简短。

3b-i) Bug fixes, Downtimes, Content Changes

Bug fixes, Downtimes, Content Changes: ehealth systems are often dynamic systems. A description of changes to methods therefore also includes important changes made on the intervention or comparator during the trial (e.g., major bug fixes or changes in the functionality or content) (5-iii) and other “unexpected events” that may have influenced study design such as staff changes, system failures/downtimes, etc. [2].

	1	2	3	4	5	
subitem not at all important	○	○	○	○	○	essential

Does your paper address subitem 3b-i?

Copy and paste relevant sections from the manuscript (include quotes in quotation marks "like this" to indicate direct quotes from your manuscript), or elaborate on this item by providing additional information not in the ms, or briefly explain why the item is not applicable/relevant for your study

您的回答

4a) Eligibility criteria for participants

Does your paper address CONSORT subitem 4a? *

Copy and paste relevant sections from the manuscript (include quotes in quotation marks "like this" to indicate direct quotes from your manuscript), or elaborate on this item by providing additional information not in the ms, or briefly explain why the item is not applicable/relevant for your study

Like this:"The staff at the community health service center recruited eligible hypertensive patients within their jurisdiction through phone calls or verbal invitations using convenience sampling method. Inclusion criteria for participants were as follows: (1) diagnosed with primary hypertension or currently taking antihypertensive medication; (2) aged over 60; and (3) proficient in using smartphones and the WeChat application. Exclusion criteria were as follows: (1) hypertensive patients undergoing non-pharmacological treatment; (2) those with diabetes, kidney disease, or other conditions requiring special dietary and exercise considerations; (3) individuals participating in or having participated in other health management projects; and (4) those unable to measure blood pressure in a home environment."

您的回复过长。请尝试让您的部分回答更简短。

4a-i) Computer / Internet literacy

Computer / Internet literacy is often an implicit “de facto” eligibility criterion - this should be explicitly clarified.

	1	2	3	4	5	
subitem not at all important	○	○	○	○	○	essential

Does your paper address subitem 4a-i?

Copy and paste relevant sections from the manuscript (include quotes in quotation marks "like this" to indicate direct quotes from your manuscript), or elaborate on this item by providing additional information not in the ms, or briefly explain why the item is not applicable/relevant for your study

您的回答

4a-ii) Open vs. closed, web-based vs. face-to-face assessments:

Open vs. closed, web-based vs. face-to-face assessments: Mention how participants were recruited (online vs. offline), e.g., from an open access website or from a clinic, and clarify if this was a purely web-based trial, or there were face-to-face components (as part of the intervention or for assessment), i.e., to what degree got the study team to know the participant. In online-only trials, clarify if participants were quasi-anonymous and whether having multiple identities was possible or whether technical or logistical measures (e.g., cookies, email confirmation, phone calls) were used to detect/prevent these.

	1	2	3	4	5	
subitem not at all important	○	○	○	○	○	essential

您的回复过长。请尝试让您的部分回答更简短。

Does your paper address subitem 4a-ii? *

Copy and paste relevant sections from the manuscript (include quotes in quotation marks "like this" to indicate direct quotes from your manuscript), or elaborate on this item by providing additional information not in the ms, or briefly explain why the item is not applicable/relevant for your study

Like this:"The staff at the community health service center recruited eligible hypertensive patients within their jurisdiction through phone calls or verbal invitations using convenience sampling method. "; "An independent data manager conducted weekly checks on the database to ensure its integrity and security. The implementation of data lockup aims to prevent any post-modification. All exported data must undergo anonymization by the data manager before statistical analysis can be conducted to safeguard the participants' information." ;"Prior to randomization, a researcher unfamiliar with the experimental design employed SPSS software (version 23; IBM Corporation) to generate 68 random numbers. Subsequently, using the visual binning method, these numbers were divided into two groups (one for the experimental group and the other for the control group). The paper slips containing the labeled random numbers were placed into sealed envelopes, and another researcher unaware of the experimental design was responsible for assigning participants to either the experimental or control group.This researcher sequentially opened the envelopes and assigned participants to the experimental or control group based on the numbers. Additionally, during the data collection phases before and after the experiment, we trained and employed two nursing graduate students unfamiliar with the study design. The data collection for the experimental group intervention process was conducted by five nursing undergraduates who were also unfamiliar with the experimental design."

4a-iii) Information giving during recruitment

Information given during recruitment. Specify how participants were briefed for recruitment and in the informed consent procedures (e.g., publish the informed consent documentation as appendix, see also item X26), as this information may have an effect on user self-selection, user expectation and may also bias results.

	1	2	3	4	5	
subitem not at all important	○	○	○	○	○	essential

您的回复过长。请尝试让您的部分回答更简短。

Does your paper address subitem 4a-iii?

Copy and paste relevant sections from the manuscript (include quotes in quotation marks "like this" to indicate direct quotes from your manuscript), or elaborate on this item by providing additional information not in the ms, or briefly explain why the item is not applicable/relevant for your study

您的回答

4b) Settings and locations where the data were collected

Does your paper address CONSORT subitem 4b? *

Copy and paste relevant sections from the manuscript (include quotes in quotation marks "like this" to indicate direct quotes from your manuscript), or elaborate on this item by providing additional information not in the ms, or briefly explain why the item is not applicable/relevant for your study

Like this:"The trial is conducted at two community health centers: Sanxiao Kou Community Health Service Center and Dongfeng Community Health Service Center, both located in Anhui province. "

4b-i) Report if outcomes were (self-)assessed through online questionnaires

Clearly report if outcomes were (self-)assessed through online questionnaires (as common in web-based trials) or otherwise.

	1	2	3	4	5	
subitem not at all important	○	○	○	○	○	essential

您的回复过长。请尝试让您的部分回答更简短。

Does your paper address subitem 4b-i? *

Copy and paste relevant sections from the manuscript (include quotes in quotation marks "like this" to indicate direct quotes from your manuscript), or elaborate on this item by providing additional information not in the ms, or briefly explain why the item is not applicable/relevant for your study

Like this:"Five undergraduate students, unfamiliar with the experimental design, collected adherence indicators from the experimental group participants weekly through WeChat. These indicators included Exercise Adherence (EA), calculated as the ratio of actual weekly exercise time meeting the prescribed intensity to the total prescribed weekly exercise time; Dietary Adherence (DA), assessed by the weekly average score of the simplified DASH grading diet index score (see protocol paper for details[35]); Medication Adherence (MA), assessed by the weekly average score of the Modified Morisky Scale (Chinese version-MMS-8, Certificate Number: 8538-1877-1559-6025-5310); and Blood Pressure Monitoring Adherence (BPMA), calculated as the ratio of actual weekly blood pressure monitoring numbers to the total recommended weekly numbers. "; "Secondary outcomes were assessed before and after the intervention. Heart rate and SEVR were measured using the cardiovascular function monitor; baPWV was measured using an arterial stiffness monitor (BX-AS-100, Intelligent Machine Institute); weight was measured using a body composition analyzer (BX-BCA-100, Intelligent Machine Institute); and lifestyle was assessed through an online questionnaire administered to participants pre- and post-intervention. "

4b-ii) Report how institutional affiliations are displayed

Report how institutional affiliations are displayed to potential participants [on ehealth media], as affiliations with prestigious hospitals or universities may affect volunteer rates, use, and reactions with regards to an intervention.(Not a required item – describe only if this may bias results)

	1	2	3	4	5	
subitem not at all important	○	○	○	○	○	essential

Does your paper address subitem 4b-ii?

Copy and paste relevant sections from the manuscript (include quotes in quotation marks "like this" to indicate direct quotes from your manuscript), or elaborate on this item by providing additional information not in the ms, or briefly explain why the item is not applicable/relevant for your study

[illegible]

您的回复过长。请尝试让您的部分回答更简短。

5) The interventions for each group with sufficient details to allow replication, including how and when they were actually administered

5-i) Mention names, credential, affiliations of the developers, sponsors, and owners

Mention names, credential, affiliations of the developers, sponsors, and owners [6] (if authors/evaluators are owners or developer of the software, this needs to be declared in a “Conflict of interest” section or mentioned elsewhere in the manuscript).

	1	2	3	4	5	
subitem not at all important	○	○	○	○	○	essential

Does your paper address subitem 5-i?

Copy and paste relevant sections from the manuscript (include quotes in quotation marks "like this" to indicate direct quotes from your manuscript), or elaborate on this item by providing additional information not in the ms, or briefly explain why the item is not applicable/relevant for your study

您的回答

5-ii) Describe the history/development process

Describe the history/development process of the application and previous formative evaluations (e.g., focus groups, usability testing), as these will have an impact on adoption/use rates and help with interpreting results.

	1	2	3	4	5	
subitem not at all important	○	○	○	○	○	essential

您的回复过长。请尝试让您的部分回答更简短。

Does your paper address subitem 5-ii?

Copy and paste relevant sections from the manuscript (include quotes in quotation marks "like this" to indicate direct quotes from your manuscript), or elaborate on this item by providing additional information not in the ms, or briefly explain why the item is not applicable/relevant for your study

您的回答

5-iii) Revisions and updating

Revisions and updating. Clearly mention the date and/or version number of the application/intervention (and comparator, if applicable) evaluated, or describe whether the intervention underwent major changes during the evaluation process, or whether the development and/or content was “frozen” during the trial. Describe dynamic components such as news feeds or changing content which may have an impact on the replicability of the intervention (for unexpected events see item 3b).

	1	2	3	4	5	
subitem not at all important	○	○	○	○	○	essential

Does your paper address subitem 5-iii?

Copy and paste relevant sections from the manuscript (include quotes in quotation marks "like this" to indicate direct quotes from your manuscript), or elaborate on this item by providing additional information not in the ms, or briefly explain why the item is not applicable/relevant for your study

您的回答

5-iv) Quality assurance methods

Provide information on quality assurance methods to ensure accuracy and quality of information provided [1], if applicable.

	1	2	3	4	5	
subitem not at all important	○	○	○	○	○	essential

您的回复过长。请尝试让您的部分回答更简短。

Does your paper address subitem 5-iv?

Copy and paste relevant sections from the manuscript (include quotes in quotation marks "like this" to indicate direct quotes from your manuscript), or elaborate on this item by providing additional information not in the ms, or briefly explain why the item is not applicable/relevant for your study

您的回答

5-v) Ensure replicability by publishing the source code, and/or providing screenshots/screen-capture video, and/or providing flowcharts of the algorithms used

Ensure replicability by publishing the source code, and/or providing screenshots/screen-capture video, and/or providing flowcharts of the algorithms used. Replicability (i.e., other researchers should in principle be able to replicate the study) is a hallmark of scientific reporting.

	1	2	3	4	5	
subitem not at all important	○	○	○	○	○	essential

Does your paper address subitem 5-v?

Copy and paste relevant sections from the manuscript (include quotes in quotation marks "like this" to indicate direct quotes from your manuscript), or elaborate on this item by providing additional information not in the ms, or briefly explain why the item is not applicable/relevant for your study

您的回答

您的回复过长。请尝试让您的部分回答更简短。

5-vi) Digital preservation

Digital preservation: Provide the URL of the application, but as the intervention is likely to change or disappear over the course of the years; also make sure the intervention is archived (Internet Archive, webcitation.org, and/or publishing the source code or screenshots/videos alongside the article). As pages behind login screens cannot be archived, consider creating demo pages which are accessible without login.

	1	2	3	4	5	
subitem not at all important	○	○	○	○	○	essential

Does your paper address subitem 5-vi?

Copy and paste relevant sections from the manuscript (include quotes in quotation marks "like this" to indicate direct quotes from your manuscript), or elaborate on this item by providing additional information not in the ms, or briefly explain why the item is not applicable/relevant for your study

您的回答

5-vii) Access

Access: Describe how participants accessed the application, in what setting/context, if they had to pay (or were paid) or not, whether they had to be a member of specific group. If known, describe how participants obtained “access to the platform and Internet” [1]. To ensure access for editors/reviewers/readers, consider to provide a “backdoor” login account or demo mode for reviewers/readers to explore the application (also important for archiving purposes, see vi).

	1	2	3	4	5	
subitem not at all important	○	○	○	○	○	essential

您的回复过长。请尝试让您的部分回答更简短。

Does your paper address subitem 5-vii? *

Copy and paste relevant sections from the manuscript (include quotes in quotation marks "like this" to indicate direct quotes from your manuscript), or elaborate on this item by providing additional information not in the ms, or briefly explain why the item is not applicable/relevant for your study

Like this:"WeChat, China's predominant social communication mobile application, serves as one of platforms for mHealth interventions. It boasts a staggering daily user count of up to 902 million and over 1 billion monthly active users spanning all age groups[17]."; "This system is installed in the Intelligent Health Cabin at community health service centers (see Figure 3). It currently supports a range of connected instruments, including cardiovascular function monitors, arteriosclerosis detectors, body composition monitors, bone densitometers, and physical fitness detectors. After completing assessments with these instruments, the system sends the collected data to its central cloud platform database. Residents need to continue to fill out various questionnaires, such as chronic disease history questionnaires, medication profiles, and family medical history surveys. Then, the system uses both instrument data and questionnaire responses to activate the intelligent decision-making and inference engine, which generates comprehensive reports. These reports provide a comprehensive evaluation of an individual's health status and offer personalized, evidence-based recommendations, namely, personalized intervention plans. "

5-viii) Mode of delivery, features/functionalities/components of the intervention and comparator, and the theoretical framework

Describe mode of delivery, features/functionalities/components of the intervention and comparator, and the theoretical framework [6] used to design them (instructional strategy [1], behaviour change techniques, persuasive features, etc., see e.g., [7, 8] for terminology). This includes an in-depth description of the content (including where it is coming from and who developed it) [1],” whether [and how] it is tailored to individual circumstances and allows users to track their progress and receive feedback” [6]. This also includes a description of communication delivery channels and – if computer-mediated communication is a component – whether communication was synchronous or asynchronous [6]. It also includes information on presentation strategies [1], including page design principles, average amount of text on pages, presence of hyperlinks to other resources, etc. [1].

	1	2	3	4	5	
subitem not at all important	○	○	○	○	○	essential

您的回复过长。请尝试让您的部分回答更简短。

Does your paper address subitem 5-viii? *

Copy and paste relevant sections from the manuscript (include quotes in quotation marks "like this" to indicate direct quotes from your manuscript), or elaborate on this item by providing additional information not in the ms, or briefly explain why the item is not applicable/relevant for your study

Like this:"We utilized the systematic workflow of the Behavior Change Wheel (BCW) to identify appropriate intervention categories and suitable Behavior Change Techniques (BCTs). Details of this process have been published in other papers[35]. Based on the identified BCTs, we developed corresponding textual content, which, along with the previously mentioned Digital Health Education Materials, collectively forms the foundational elements of the Digital Media Intervention for Change (DMIC) theory. Subsequently, additional elements of the DMIC theory, namely proximal assessment indicators and decision rules, were determined based on literature review and expert opinions."; "The Intelligent Health Promotion System is a cloud platform-based system that leverages health sign data and health questionnaire responses to generate intelligent health reports, personalized exercise prescriptions, and other tailored health recommendations. It also tracks and monitors individual health data. This system comprises three main layers: the perception layer, the decision layer, and the application layer (see Figure 2)."; "This system is installed in the Intelligent Health Cabin at community health service centers (see Figure 3). It currently supports a range of connected instruments, including cardiovascular function monitors, arteriosclerosis detectors, body composition monitors, bone densitometers, and physical fitness detectors. After completing assessments with these instruments, the system sends the collected data to its central cloud platform database. Residents need to continue to fill out various questionnaires, such as chronic disease history questionnaires, medication profiles, and family medical history surveys. Then, the system uses both instrument data and questionnaire responses to activate the intelligent decision-making and inference engine, which generates comprehensive reports. These reports provide a comprehensive evaluation of an individual's health status and offer personalized, evidence-based recommendations, namely, personalized intervention plans. "

5-ix) Describe use parameters

Describe use parameters (e.g., intended “doses” and optimal timing for use). Clarify what instructions or recommendations were given to the user, e.g., regarding timing, frequency, heaviness of use, if any, or was the intervention used ad libitum.

	1	2	3	4	5	
subitem not at all important	○	○	○	○	○	essential

您的回复过长。请尝试让您的部分回答更简短。

Does your paper address subitem 5-ix?

Copy and paste relevant sections from the manuscript (include quotes in quotation marks "like this" to indicate direct quotes from your manuscript), or elaborate on this item by providing additional information not in the ms, or briefly explain why the item is not applicable/relevant for your study

您的回答

5-x) Clarify the level of human involvement

Clarify the level of human involvement (care providers or health professionals, also technical assistance) in the e-intervention or as co-intervention (detail number and expertise of professionals involved, if any, as well as “type of assistance offered, the timing and frequency of the support, how it is initiated, and the medium by which the assistance is delivered”. It may be necessary to distinguish between the level of human involvement required for the trial, and the level of human involvement required for a routine application outside of a RCT setting (discuss under item 21 – generalizability).

	1	2	3	4	5	
subitem not at all important	○	○	○	○	○	essential

Does your paper address subitem 5-x?

Copy and paste relevant sections from the manuscript (include quotes in quotation marks "like this" to indicate direct quotes from your manuscript), or elaborate on this item by providing additional information not in the ms, or briefly explain why the item is not applicable/relevant for your study

您的回答

您的回复过长。请尝试让您的部分回答更简短。

5-xi) Report any prompts/reminders used

Report any prompts/reminders used: Clarify if there were prompts (letters, emails, phone calls, SMS) to use the application, what triggered them, frequency etc. It may be necessary to distinguish between the level of prompts/reminders required for the trial, and the level of prompts/reminders for a routine application outside of a RCT setting (discuss under item 21 – generalizability).

	1	2	3	4	5	
subitem not at all important	○	○	○	○	○	essential

Does your paper address subitem 5-xi? *

Copy and paste relevant sections from the manuscript (include quotes in quotation marks "like this" to indicate direct quotes from your manuscript), or elaborate on this item by providing additional information not in the ms, or briefly explain why the item is not applicable/relevant for your study

Like this:"The study protocol was previously published[35]. This was a randomized controlled trial. The experimental group received health behavioral digital intervention based on an intelligent health promotion system and WeChat for 12 weeks, while The control group received routine health services and was provided with a "Hypertension Self-Management Manual" to guide daily health behaviors (refer to Figure 9)."

5-xii) Describe any co-interventions (incl. training/support)

Describe any co-interventions (incl. training/support): Clearly state any interventions that are provided in addition to the targeted eHealth intervention, as ehealth intervention may not be designed as stand-alone intervention. This includes training sessions and support [1]. It may be necessary to distinguish between the level of training required for the trial, and the level of training for a routine application outside of a RCT setting (discuss under item 21 – generalizability.

	1	2	3	4	5	
subitem not at all important	○	○	○	○	○	essential

您的回复过长。请尝试让您的部分回答更简短。

Does your paper address subitem 5-xii? *

Copy and paste relevant sections from the manuscript (include quotes in quotation marks "like this" to indicate direct quotes from your manuscript), or elaborate on this item by providing additional information not in the ms, or briefly explain why the item is not applicable/relevant for your study

Like this:"Additionally, during the data collection phases before and after the experiment, we trained and employed two nursing graduate students unfamiliar with the study design. The data collection for the experimental group intervention process was conducted by five nursing undergraduates who were also unfamiliar with the experimental design."

6a) Completely defined pre-specified primary and secondary outcome measures, including how and when they were assessed

您的回复过长。请尝试让您的部分回答更简短。

Does your paper address CONSORT subitem 6a? *

Copy and paste relevant sections from the manuscript (include quotes in quotation marks "like this" to indicate direct quotes from your manuscript), or elaborate on this item by providing additional information not in the ms, or briefly explain why the item is not applicable/relevant for your study

Like this:"Primary outcomes
Before and 12 weeks after the intervention, we utilized a cardiovascular function monitoring device (BX-CFTI-100, Intelligent Machine Institute) to measure participants' blood pressure. Measurement details have been published[13]. Five undergraduate students, unfamiliar with the experimental design, collected adherence indicators from the experimental group participants weekly through WeChat. These indicators included Exercise Adherence (EA), calculated as the ratio of actual weekly exercise time meeting the prescribed intensity to the total prescribed weekly exercise time; Dietary Adherence (DA), assessed by the weekly average score of the simplified DASH grading diet index score (see protocol paper for details[35]); Medication Adherence (MA), assessed by the weekly average score of the Modified Morisky Scale (Chinese version-MMS-8, Certificate Number: 8538-1877-1559-6025-5310); and Blood Pressure Monitoring Adherence (BPMA), calculated as the ratio of actual weekly blood pressure monitoring numbers to the total recommended weekly numbers. "; "Secondary outcomes
Secondary outcomes were assessed before and after the intervention. Heart rate and SEVR were measured using the cardiovascular function monitor; baPWV was measured using an arterial stiffness monitor (BX-AS-100, Intelligent Machine Institute); weight was measured using a body composition analyzer (BX-BCA-100, Intelligent Machine Institute); and lifestyle was assessed through an online questionnaire administered to participants pre- and post-intervention. Participants were questioned about health-related behaviors, including the frequency of smoking, drinking, diet, and physical activity. For smoking, the lifelong smoking amount of participants was calculated based on the quantity and weekly frequency of cigarettes smoked. For those who had quit smoking, this calculation also included their smoking history before quitting.The alcohol content in one bottle of the most popular alcoholic beverages in Anhui province is as follows: beer (500 ml, 3.2% alcohol), 17.5 g; white liquor (450 ml, 42% alcohol), 210 g; and wine (750 ml, 13.5%–14% alcohol), 97.5 g. Daily alcohol consumption was calculated using these values. Missing values for smoking and drinking data were set to zero. Participants were asked about the types, durations (in minutes), and frequencies (per week) of physical activities they engaged in. Physical activity time was determined in minutes/MET/day based on activity codes and metabolic equivalent (MET) intensities in the "Compendium of Physical Activities." Weekly Exercise time (MET-min/week) and weekly Physical activity time (MET-min/week) were calculated, with missing values set to the median[37]."

您的回复过长。请尝试让您的部分回答更简短。

6a-i) Online questionnaires: describe if they were validated for online use and apply CHERRIES items to describe how the questionnaires were designed/deployed

If outcomes were obtained through online questionnaires, describe if they were validated for online use and apply CHERRIES items to describe how the questionnaires were designed/deployed [9].

	1	2	3	4	5	
subitem not at all important	○	○	○	○	○	essential

Does your paper address subitem 6a-i?

Copy and paste relevant sections from manuscript text

您的回答

6a-ii) Describe whether and how “use” (including intensity of use/dosage) was defined/measured/monitored

Describe whether and how “use” (including intensity of use/dosage) was defined/measured/monitored (logins, logfile analysis, etc.). Use/adoption metrics are important process outcomes that should be reported in any ehealth trial.

	1	2	3	4	5	
subitem not at all important	○	○	○	○	○	essential

Does your paper address subitem 6a-ii?

Copy and paste relevant sections from manuscript text

您的回答

您的回复过长。请尝试让您的部分回答更简短。

6a-iii) Describe whether, how, and when qualitative feedback from participants was obtained

Describe whether, how, and when qualitative feedback from participants was obtained (e.g., through emails, feedback forms, interviews, focus groups).

	1	2	3	4	5	
subitem not at all important	○	○	○	○	○	essential

Does your paper address subitem 6a-iii?

Copy and paste relevant sections from manuscript text

您的回答

6b) Any changes to trial outcomes after the trial commenced, with reasons

Does your paper address CONSORT subitem 6b? *

Copy and paste relevant sections from the manuscript (include quotes in quotation marks "like this" to indicate direct quotes from your manuscript), or elaborate on this item by providing additional information not in the ms, or briefly explain why the item is not applicable/relevant for your study

None. No changes were made to the test results after the start of the test.

7a) How sample size was determined

NPT: When applicable, details of whether and how the clustering by care provides or centers was addressed

您的回复过长。请尝试让您的部分回答更简短。

7a-i) Describe whether and how expected attrition was taken into account when calculating the sample size

Describe whether and how expected attrition was taken into account when calculating the sample size.

	1	2	3	4	5	
subitem not at all important	○	○	○	○	○	essential

Does your paper address subitem 7a-i?

Copy and paste relevant sections from manuscript title (include quotes in quotation marks "like this" to indicate direct quotes from your manuscript), or elaborate on this item by providing additional information not in the ms, or briefly explain why the item is not applicable/relevant for your study

您的回答

7b) When applicable, explanation of any interim analyses and stopping guidelines

Does your paper address CONSORT subitem 7b? *

Copy and paste relevant sections from the manuscript (include quotes in quotation marks "like this" to indicate direct quotes from your manuscript), or elaborate on this item by providing additional information not in the ms, or briefly explain why the item is not applicable/relevant for your study

None. This is not applicable for this study.

8a) Method used to generate the random allocation sequence

NPT: When applicable, how care providers were allocated to each trial group

您的回复过长。请尝试让您的部分回答更简短。

Does your paper address CONSORT subitem 8a? *

Copy and paste relevant sections from the manuscript (include quotes in quotation marks "like this" to indicate direct quotes from your manuscript), or elaborate on this item by providing additional information not in the ms, or briefly explain why the item is not applicable/relevant for your study

Like this:"Prior to randomization, a researcher unfamiliar with the experimental design employed SPSS software (version 23; IBM Corporation) to generate 68 random numbers. Subsequently, using the visual binning method, these numbers were divided into two groups (one for the experimental group and the other for the control group). The paper slips containing the labeled random numbers were placed into sealed envelopes, and another researcher unaware of the experimental design was responsible for assigning participants to either the experimental or control group.This researcher sequentially opened the envelopes and assigned participants to the experimental or control group based on the numbers. "

8b) Type of randomisation; details of any restriction (such as blocking and block size)

Does your paper address CONSORT subitem 8b? *

Copy and paste relevant sections from the manuscript (include quotes in quotation marks "like this" to indicate direct quotes from your manuscript), or elaborate on this item by providing additional information not in the ms, or briefly explain why the item is not applicable/relevant for your study

Like this:"Prior to randomization, a researcher unfamiliar with the experimental design employed SPSS software (version 23; IBM Corporation) to generate 68 random numbers. Subsequently, using the visual binning method, these numbers were divided into two groups (one for the experimental group and the other for the control group). The paper slips containing the labeled random numbers were placed into sealed envelopes, and another researcher unaware of the experimental design was responsible for assigning participants to either the experimental or control group.This researcher sequentially opened the envelopes and assigned participants to the experimental or control group based on the numbers. "

9) Mechanism used to implement the random allocation sequence (such as sequentially numbered containers), describing any steps taken to conceal the sequence until interventions were assigned

您的回复过长。请尝试让您的部分回答更简短。

Does your paper address CONSORT subitem 9? *

Copy and paste relevant sections from the manuscript (include quotes in quotation marks "like this" to indicate direct quotes from your manuscript), or elaborate on this item by providing additional information not in the ms, or briefly explain why the item is not applicable/relevant for your study

Like this:"Prior to randomization, a researcher unfamiliar with the experimental design employed SPSS software (version 23; IBM Corporation) to generate 68 random numbers. Subsequently, using the visual binning method, these numbers were divided into two groups (one for the experimental group and the other for the control group). The paper slips containing the labeled random numbers were placed into sealed envelopes, and another researcher unaware of the experimental design was responsible for assigning participants to either the experimental or control group.This researcher sequentially opened the envelopes and assigned participants to the experimental or control group based on the numbers. "

10) Who generated the random allocation sequence, who enrolled participants, and who assigned participants to interventions

Does your paper address CONSORT subitem 10? *

Copy and paste relevant sections from the manuscript (include quotes in quotation marks "like this" to indicate direct quotes from your manuscript), or elaborate on this item by providing additional information not in the ms, or briefly explain why the item is not applicable/relevant for your study

Like this:"Prior to randomization, a researcher unfamiliar with the experimental design employed SPSS software (version 23; IBM Corporation) to generate 68 random numbers. Subsequently, using the visual binning method, these numbers were divided into two groups (one for the experimental group and the other for the control group). The paper slips containing the labeled random numbers were placed into sealed envelopes, and another researcher unaware of the experimental design was responsible for assigning participants to either the experimental or control group.This researcher sequentially opened the envelopes and assigned participants to the experimental or control group based on the numbers. "

11a) If done, who was blinded after assignment to interventions (for example, participants, care providers, those assessing outcomes) and how

NPT: Whether or not administering co-interventions were blinded to group assignment

您的回复过长。请尝试让您的部分回答更简短。

11a-i) Specify who was blinded, and who wasn’t

Specify who was blinded, and who wasn’t. Usually, in web-based trials it is not possible to blind the participants [1, 3] (this should be clearly acknowledged), but it may be possible to blind outcome assessors, those doing data analysis or those administering co-interventions (if any).

	1	2	3	4	5	
subitem not at all important	○	○	○	○	○	essential

Does your paper address subitem 11a-i? *

Copy and paste relevant sections from the manuscript (include quotes in quotation marks "like this" to indicate direct quotes from your manuscript), or elaborate on this item by providing additional information not in the ms, or briefly explain why the item is not applicable/relevant for your study

Like this:"Prior to randomization, a researcher unfamiliar with the experimental design employed SPSS software (version 23; IBM Corporation) to generate 68 random numbers. Subsequently, using the visual binning method, these numbers were divided into two groups (one for the experimental group and the other for the control group). The paper slips containing the labeled random numbers were placed into sealed envelopes, and another researcher unaware of the experimental design was responsible for assigning participants to either the experimental or control group.This researcher sequentially opened the envelopes and assigned participants to the experimental or control group based on the numbers. "

11a-ii) Discuss e.g., whether participants knew which intervention was the “intervention of interest” and which one was the “comparator”

Informed consent procedures (4a-ii) can create biases and certain expectations - discuss e.g., whether participants knew which intervention was the “intervention of interest” and which one was the “comparator”.

	1	2	3	4	5	
subitem not at all important	○	○	○	○	○	essential

您的回复过长。请尝试让您的部分回答更简短。

Does your paper address subitem 11a-ii?

Copy and paste relevant sections from the manuscript (include quotes in quotation marks "like this" to indicate direct quotes from your manuscript), or elaborate on this item by providing additional information not in the ms, or briefly explain why the item is not applicable/relevant for your study

您的回答

11b) If relevant, description of the similarity of interventions

(this item is usually not relevant for ehealth trials as it refers to similarity of a placebo or sham intervention to a active medication/intervention)

Does your paper address CONSORT subitem 11b? *

Copy and paste relevant sections from the manuscript (include quotes in quotation marks "like this" to indicate direct quotes from your manuscript), or elaborate on this item by providing additional information not in the ms, or briefly explain why the item is not applicable/relevant for your study

Like this:"The experimental group received health behavioral digital intervention based on an intelligent health promotion system and WeChat for 12 weeks, while The control group received routine health services and was provided with a "Hypertension Self-Management Manual" to guide daily health behaviors (refer to Figure 9)."

12a) Statistical methods used to compare groups for primary and secondary outcomes

NPT: When applicable, details of whether and how the clustering by care providers or centers was addressed

您的回复过长。请尝试让您的部分回答更简短。

Does your paper address CONSORT subitem 12a? *

Copy and paste relevant sections from the manuscript (include quotes in quotation marks "like this" to indicate direct quotes from your manuscript), or elaborate on this item by providing additional information not in the ms, or briefly explain why the item is not applicable/relevant for your study

Like this:"Data analysis was carried out using SPSS, with independent sample t-tests and chi-square tests employed to evaluate the significance of differences between the two groups. Paired t-tests and McNemar's tests were utilized to compare differences within the same group before and after the three-month intervention. A P-value of less than .05 was considered statistically significant."

12a-i) Imputation techniques to deal with attrition / missing values

Imputation techniques to deal with attrition / missing values: Not all participants will use the intervention/comparator as intended and attrition is typically high in ehealth trials. Specify how participants who did not use the application or dropped out from the trial were treated in the statistical analysis (a complete case analysis is strongly discouraged, and simple imputation techniques such as LOCF may also be problematic [4]).

	1	2	3	4	5	
subitem not at all important	○	○	○	○	○	essential

Does your paper address subitem 12a-i? *

Copy and paste relevant sections from the manuscript (include quotes in quotation marks "like this" to indicate direct quotes from your manuscript), or elaborate on this item by providing additional information not in the ms, or briefly explain why the item is not applicable/relevant for your study

Like this:"A total of 54 participants (30 women and 24 men; 67.24±4.19 years) were included in the final analysis: 23 in the experimental group and 31 in the control group. This exclusion was due to various reasons, including hospitalization for illness (2 individuals), inability to complete the post-assessment (3 individuals), health conditions deteriorating to the point of hindering PA (4 individuals), voluntary withdrawal from the study (4 individuals), and a change in antihypertensive medication (1 individual). Other patients did not change their hypertension medication during the intervention."

您的回复过长。请尝试让您的部分回答更简短。

12b) Methods for additional analyses, such as subgroup analyses and adjusted analyses

Does your paper address CONSORT subitem 12b? *

Copy and paste relevant sections from the manuscript (include quotes in quotation marks "like this" to indicate direct quotes from your manuscript), or elaborate on this item by providing additional information not in the ms, or briefly explain why the item is not applicable/relevant for your study

Like this:"Table 2 displays the baseline and posttest results for both groups regarding health outcomes, adherence indicators, and learning performance. Significant changes were observed in SBP, exercise time, MA, BP monitoring frequency, and learning performance in the intergroup comparison after 12 weeks, as well as in the before-and-after comparisons of both groups (P<.05). The experimental group showed improvements in subendocardial viability ratio (SEVR), recommended types and amounts of diet, compared to the control group after the intervention (P<.05). The PA time increased for the experimental group both in intergroup and before-and-after comparisons (P<.05). Both groups showed a reduction in weight after intervention (P<.05). Furthermore, the Cohen's d values reflecting effect size were greater than 0.5, for all variables except physical activity time, indicating a intermediate and above effect size. Among these variables, health outcome SEVR, recommended types of diet (e.g., occasionally, sometimes, often), recommended amount of diet (e.g., occasionally, sometimes, often), blood pressure monitoring frequency (e.g., measure daily, measure 1-3 times a week, measure whenever remembered) and learning performance had Cohen's d values greater than 0.8, suggesting a large effect size."; "The experimental group collected weekly average adherence indicators from the first week after the assessing and preparing phase (the second week of the project) through the 11th week (the twelfth week of the project), as depicted in the curve shown in Figure 10. From weeks 1 to 4, the exercise adherence (EA) curve indicated that this phase was when the intervention participants were most actively engaged in PA. They exceeded the prescribed exercise volume (>1). By week 5, the participants showed lower EA, followed by two weeks of rebound. Subsequently, EA sharply declined, reaching a stable state in weeks 10-11. Dietary adherence (DA) curve indicators exhibited the most noticeable fluctuations, reaching peaks in weeks 4 and 8, and valleys in weeks 6 and 10. MA gradually increased from weeks 1 to 4, experienced fluctuations, and then steadily declined starting from week 7. Among the four indicators, the BPMA curve had smaller fluctuations. It steadily increased from weeks 1 to 3, reached a low point in week 7, and then remained relatively stable at 0.72."

X26) REB/IRB Approval and Ethical Considerations [recommended as subheading under "Methods"] (not a CONSORT item)

您的回复过长。请尝试让您的部分回答更简短。

X26-i) Comment on ethics committee approval

	1	2	3	4	5	
subitem not at all important	○	○	○	○	○	essential

Does your paper address subitem X26-i?

Copy and paste relevant sections from the manuscript (include quotes in quotation marks "like this" to indicate direct quotes from your manuscript), or elaborate on this item by providing additional information not in the ms, or briefly explain why the item is not applicable/relevant for your study

您的回答

x26-ii) Outline informed consent procedures

Outline informed consent procedures e.g., if consent was obtained offline or online (how? Checkbox, etc.?), and what information was provided (see 4a-ii). See [6] for some items to be included in informed consent documents.

	1	2	3	4	5	
subitem not at all important	○	○	○	○	○	essential

Does your paper address subitem X26-ii?

Copy and paste relevant sections from the manuscript (include quotes in quotation marks "like this" to indicate direct quotes from your manuscript), or elaborate on this item by providing additional information not in the ms, or briefly explain why the item is not applicable/relevant for your study

您的回答

您的回复过长。请尝试让您的部分回答更简短。

X26-iii) Safety and security procedures

Safety and security procedures, incl. privacy considerations, and any steps taken to reduce the likelihood or detection of harm (e.g., education and training, availability of a hotline)

	1	2	3	4	5	
subitem not at all important	○	○	○	○	○	essential

Does your paper address subitem X26-iii?

Copy and paste relevant sections from the manuscript (include quotes in quotation marks "like this" to indicate direct quotes from your manuscript), or elaborate on this item by providing additional information not in the ms, or briefly explain why the item is not applicable/relevant for your study

您的回答

RESULTS

13a) For each group, the numbers of participants who were randomly assigned, received intended treatment, and were analysed for the primary outcome

NPT: The number of care providers or centers performing the intervention in each group and the number of patients treated by each care provider in each center

您的回复过长。请尝试让您的部分回答更简短。

Does your paper address CONSORT subitem 13a? *

Copy and paste relevant sections from the manuscript (include quotes in quotation marks "like this" to indicate direct quotes from your manuscript), or elaborate on this item by providing additional information not in the ms, or briefly explain why the item is not applicable/relevant for your study

Like this:"Between September 5, 2022, and September 19, 2022, a total of 68 hypertensive patients were recruited through phone or verbal invitations from two community health centers in Anhui province. Participants were re-invited to the community health service center for health data and information collection between December 20, 2022, and January 5, 2023. A total of 54 participants (30 women and 24 men; 67.24±4.19 years) were included in the final analysis: 23 in the experimental group and 31 in the control group. This exclusion was due to various reasons, including hospitalization for illness (2 individuals), inability to complete the post-assessment (3 individuals), health conditions deteriorating to the point of hindering PA (4 individuals), voluntary withdrawal from the study (4 individuals), and a change in antihypertensive medication (1 individual). Other patients did not change their hypertension medication during the intervention."

13b) For each group, losses and exclusions after randomisation, together with reasons

Does your paper address CONSORT subitem 13b? (NOTE: Preferably, this is shown in a CONSORT flow diagram) *

Copy and paste relevant sections from the manuscript (include quotes in quotation marks "like this" to indicate direct quotes from your manuscript), or elaborate on this item by providing additional information not in the ms, or briefly explain why the item is not applicable/relevant for your study

Like this:"Between September 5, 2022, and September 19, 2022, a total of 68 hypertensive patients were recruited through phone or verbal invitations from two community health centers in Anhui province. Participants were re-invited to the community health service center for health data and information collection between December 20, 2022, and January 5, 2023. A total of 54 participants (30 women and 24 men; 67.24±4.19 years) were included in the final analysis: 23 in the experimental group and 31 in the control group. This exclusion was due to various reasons, including hospitalization for illness (2 individuals), inability to complete the post-assessment (3 individuals), health conditions deteriorating to the point of hindering PA (4 individuals), voluntary withdrawal from the study (4 individuals), and a change in antihypertensive medication (1 individual). Other patients did not change their hypertension medication during the intervention."

您的回复过长。请尝试让您的部分回答更简短。

13b-i) Attrition diagram

Strongly recommended: An attrition diagram (e.g., proportion of participants still logging in or using the intervention/comparator in each group plotted over time, similar to a survival curve) or other figures or tables demonstrating usage/dose/engagement.

	1	2	3	4	5	
subitem not at all important	○	○	○	○	○	essential

Does your paper address subitem 13b-i?

Copy and paste relevant sections from the manuscript or cite the figure number if applicable (include quotes in quotation marks "like this" to indicate direct quotes from your manuscript), or elaborate on this item by providing additional information not in the ms, or briefly explain why the item is not applicable/relevant for your study

您的回答

14a) Dates defining the periods of recruitment and follow-up

Does your paper address CONSORT subitem 14a? *

Copy and paste relevant sections from the manuscript (include quotes in quotation marks "like this" to indicate direct quotes from your manuscript), or elaborate on this item by providing additional information not in the ms, or briefly explain why the item is not applicable/relevant for your study

Like this:"Between September 5, 2022, and September 19, 2022, a total of 68 hypertensive patients were recruited through phone or verbal invitations from two community health centers in Anhui province. Participants were re-invited to the community health service center for health data and information collection between December 20, 2022, and January 5, 2023. A total of 54 participants (30 women and 24 men; 67.24±4.19 years) were included in the final analysis: 23 in the experimental group and 31 in the control group. This exclusion was due to various reasons, including hospitalization for illness (2 individuals), inability to complete the post-assessment (3 individuals), health conditions deteriorating to the point of hindering PA (4 individuals), voluntary withdrawal from the study (4 individuals), and a change in antihypertensive medication (1 individual). Other patients did not change their hypertension medication during the intervention."

您的回复过长。请尝试让您的部分回答更简短。

14a-i) Indicate if critical “secular events” fell into the study period

Indicate if critical “secular events” fell into the study period, e.g., significant changes in Internet resources available or “changes in computer hardware or Internet delivery resources”

	1	2	3	4	5	
subitem not at all important	○	○	○	○	○	essential

Does your paper address subitem 14a-i?

Copy and paste relevant sections from the manuscript (include quotes in quotation marks "like this" to indicate direct quotes from your manuscript), or elaborate on this item by providing additional information not in the ms, or briefly explain why the item is not applicable/relevant for your study

您的回答

14b) Why the trial ended or was stopped (early)

Does your paper address CONSORT subitem 14b? *

Copy and paste relevant sections from the manuscript (include quotes in quotation marks "like this" to indicate direct quotes from your manuscript), or elaborate on this item by providing additional information not in the ms, or briefly explain why the item is not applicable/relevant for your study

None. No early termination of our study has occurred.

15) A table showing baseline demographic and clinical characteristics for each group

NPT: When applicable, a description of care providers (case volume, qualification, expertise, etc.) and centers (volume) in each group

您的回复过长。请尝试让您的部分回答更简短。

Does your paper address CONSORT subitem 15? *

Copy and paste relevant sections from the manuscript (include quotes in quotation marks "like this" to indicate direct quotes from your manuscript), or elaborate on this item by providing additional information not in the ms, or briefly explain why the item is not applicable/relevant for your study

Like this:""

15-i) Report demographics associated with digital divide issues

In ehealth trials it is particularly important to report demographics associated with digital divide issues, such as age, education, gender, social-economic status, computer/Internet/ehealth literacy of the participants, if known.

	1	2	3	4	5	
subitem not at all important	○	○	○	○	○	essential

Does your paper address subitem 15-i? *

Copy and paste relevant sections from the manuscript (include quotes in quotation marks "like this" to indicate direct quotes from your manuscript), or elaborate on this item by providing additional information not in the ms, or briefly explain why the item is not applicable/relevant for your study

Like this:"Inclusion criteria for participants were as follows: (1) diagnosed with primary hypertension or currently taking antihypertensive medication; (2) aged over 60; and (3) proficient in using smartphones and the WeChat application. Exclusion criteria were as follows: (1) hypertensive patients undergoing non-pharmacological treatment; (2) those with diabetes, kidney disease, or other conditions requiring special dietary and exercise considerations; (3) individuals participating in or having participated in other health management projects; and (4) those unable to measure blood pressure in a home environment."

16) For each group, number of participants (denominator) included in each analysis and whether the analysis was by original assigned groups

您的回复过长。请尝试让您的部分回答更简短。

16-i) Report multiple “denominators” and provide definitions

Report multiple “denominators” and provide definitions: Report N’s (and effect sizes) “across a range of study participation [and use] thresholds” [1], e.g., N exposed, N consented, N used more than x times, N used more than y weeks, N participants “used” the intervention/comparator at specific pre-defined time points of interest (in absolute and relative numbers per group). Always clearly define “use” of the intervention.

	1	2	3	4	5	
subitem not at all important	○	○	○	○	○	essential

Does your paper address subitem 16-i? *

Copy and paste relevant sections from the manuscript (include quotes in quotation marks "like this" to indicate direct quotes from your manuscript), or elaborate on this item by providing additional information not in the ms, or briefly explain why the item is not applicable/relevant for your study

Like this:""

16-ii) Primary analysis should be intent-to-treat

Primary analysis should be intent-to-treat, secondary analyses could include comparing only “users”, with the appropriate caveats that this is no longer a randomized sample (see 18-i).

	1	2	3	4	5	
subitem not at all important	○	○	○	○	○	essential

Does your paper address subitem 16-ii?

Copy and paste relevant sections from the manuscript (include quotes in quotation marks "like this" to indicate direct quotes from your manuscript), or elaborate on this item by providing additional information not in the ms, or briefly explain why the item is not applicable/relevant for your study

您的回答

您的回复过长。请尝试让您的部分回答更简短。

17a) For each primary and secondary outcome, results for each group, and the estimated effect size and its precision (such as 95% confidence interval)

Does your paper address CONSORT subitem 17a? *

Copy and paste relevant sections from the manuscript (include quotes in quotation marks "like this" to indicate direct quotes from your manuscript), or elaborate on this item by providing additional information not in the ms, or briefly explain why the item is not applicable/relevant for your study

Like this:"Table 2 displays the baseline and posttest results for both groups regarding health outcomes, adherence indicators, and learning performance. Significant changes were observed in SBP, exercise time, MA, BP monitoring frequency, and learning performance in the intergroup comparison after 12 weeks, as well as in the before-and-after comparisons of both groups (P<.05). The experimental group showed improvements in subendocardial viability ratio (SEVR), recommended types and amounts of diet, compared to the control group after the intervention (P<.05). The PA time increased for the experimental group both in intergroup and before-and-after comparisons (P<.05). Both groups showed a reduction in weight after intervention (P<.05). Furthermore, the Cohen's d values reflecting effect size were greater than 0.5, for all variables except physical activity time, indicating a intermediate and above effect size. Among these variables, health outcome SEVR, recommended types of diet (e.g., occasionally, sometimes, often), recommended amount of diet (e.g., occasionally, sometimes, often), blood pressure monitoring frequency (e.g., measure daily, measure 1-3 times a week, measure whenever remembered) and learning performance had Cohen's d values greater than 0.8, suggesting a large effect size."

17a-i) Presentation of process outcomes such as metrics of use and intensity of use

In addition to primary/secondary (clinical) outcomes, the presentation of process outcomes such as metrics of use and intensity of use (dose, exposure) and their operational definitions is critical. This does not only refer to metrics of attrition (13-b) (often a binary variable), but also to more continuous exposure metrics such as “average session length”. These must be accompanied by a technical description how a metric like a “session” is defined (e.g., timeout after idle time) [1] (report under item 6a).

	1	2	3	4	5	
subitem not at all important	○	○	○	○	○	essential

您的回复过长。请尝试让您的部分回答更简短。

Does your paper address subitem 17a-i?

Copy and paste relevant sections from the manuscript (include quotes in quotation marks "like this" to indicate direct quotes from your manuscript), or elaborate on this item by providing additional information not in the ms, or briefly explain why the item is not applicable/relevant for your study

您的回答

17b) For binary outcomes, presentation of both absolute and relative effect sizes is recommended

Does your paper address CONSORT subitem 17b? *

Copy and paste relevant sections from the manuscript (include quotes in quotation marks "like this" to indicate direct quotes from your manuscript), or elaborate on this item by providing additional information not in the ms, or briefly explain why the item is not applicable/relevant for your study

Like this:"Table 2. Intervention of HBDIHP effects (N=54)."

18) Results of any other analyses performed, including subgroup analyses and adjusted analyses, distinguishing pre-specified from exploratory

您的回复过长。请尝试让您的部分回答更简短。

Does your paper address CONSORT subitem 18? *

Copy and paste relevant sections from the manuscript (include quotes in quotation marks "like this" to indicate direct quotes from your manuscript), or elaborate on this item by providing additional information not in the ms, or briefly explain why the item is not applicable/relevant for your study

Like this:"The observations suggest that our program may have improved specific health outcomes and adherence to health behaviors in elderly hypertensive patients. In terms of health outcomes, participants observed significant improvements in SBP, SEVR, and weight. Moreover, there were noteworthy changes in adherence indicators, such as exercise duration, medication adherence, physical activity duration, frequency of blood pressure monitoring, and learning performance. However, due to our small sample size and short intervention duration, a larger sample size and longer randomized controlled trial are needed to validate the intervention's effects, explore its mechanisms, and identify the specific design elements that are effective. Additionally, among the four adherence behaviors, DA is the most susceptible to external influences, and more BCTs targeting DA should be considered in intervention design."

18-i) Subgroup analysis of comparing only users

A subgroup analysis of comparing only users is not uncommon in ehealth trials, but if done, it must be stressed that this is a self-selected sample and no longer an unbiased sample from a randomized trial (see 16-iii).

	1	2	3	4	5	
subitem not at all important	○	○	○	○	○	essential

Does your paper address subitem 18-i?

Copy and paste relevant sections from the manuscript (include quotes in quotation marks "like this" to indicate direct quotes from your manuscript), or elaborate on this item by providing additional information not in the ms, or briefly explain why the item is not applicable/relevant for your study

您的回答

19) All important harms or unintended effects in each group

(for specific guidance see CONSORT for harms)

您的回复过长。请尝试让您的部分回答更简短。

Does your paper address CONSORT subitem 19? *

Copy and paste relevant sections from the manuscript (include quotes in quotation marks "like this" to indicate direct quotes from your manuscript), or elaborate on this item by providing additional information not in the ms, or briefly explain why the item is not applicable/relevant for your study

None.

19-i) Include privacy breaches, technical problems

Include privacy breaches, technical problems. This does not only include physical “harm” to participants, but also incidents such as perceived or real privacy breaches [1], technical problems, and other unexpected/unintended incidents. “Unintended effects” also includes unintended positive effects [2].

	1	2	3	4	5	
subitem not at all important	○	○	○	○	○	essential

Does your paper address subitem 19-i?

Copy and paste relevant sections from the manuscript (include quotes in quotation marks "like this" to indicate direct quotes from your manuscript), or elaborate on this item by providing additional information not in the ms, or briefly explain why the item is not applicable/relevant for your study

您的回答

19-ii) Include qualitative feedback from participants or observations from staff/researchers

Include qualitative feedback from participants or observations from staff/researchers, if available, on strengths and shortcomings of the application, especially if they point to unintended/unexpected effects or uses. This includes (if available) reasons for why people did or did not use the application as intended by the developers.

1	2	3	4	5

您的回复过长。请尝试让您的部分回答更简短。

Does your paper address subitem 19-ii?

Copy and paste relevant sections from the manuscript (include quotes in quotation marks "like this" to indicate direct quotes from your manuscript), or elaborate on this item by providing additional information not in the ms, or briefly explain why the item is not applicable/relevant for your study

您的回答

DISCUSSION

22) Interpretation consistent with results, balancing benefits and harms, and considering other relevant evidence

NPT: In addition, take into account the choice of the comparator, lack of or partial blinding, and unequal expertise of care providers or centers in each group

22-i) Restate study questions and summarize the answers suggested by the data, starting with primary outcomes and process outcomes (use)

Restate study questions and summarize the answers suggested by the data, starting with primary outcomes and process outcomes (use).

	1	2	3	4	5	
subitem not at all important	○	○	○	○	○	essential

您的回复过长。请尝试让您的部分回答更简短。

Does your paper address subitem 22-i? *

Copy and paste relevant sections from the manuscript (include quotes in quotation marks "like this" to indicate direct quotes from your manuscript), or elaborate on this item by providing additional information not in the ms, or briefly explain why the item is not applicable/relevant for your study

Like this:"The observations suggest that our program may have improved specific health outcomes and adherence to health behaviors in elderly hypertensive patients. In terms of health outcomes, participants observed significant improvements in SBP, SEVR, and weight. Moreover, there were noteworthy changes in adherence indicators, such as exercise duration, medication adherence, physical activity duration, frequency of blood pressure monitoring, and learning performance. However, due to our small sample size and short intervention duration, a larger sample size and longer randomized controlled trial are needed to validate the intervention's effects, explore its mechanisms, and identify the specific design elements that are effective. Additionally, among the four adherence behaviors, DA is the most susceptible to external influences, and more BCTs targeting DA should be considered in intervention design."

22-ii) Highlight unanswered new questions, suggest future research

Highlight unanswered new questions, suggest future research.

	1	2	3	4	5	
subitem not at all important	○	○	○	○	○	essential

Does your paper address subitem 22-ii?

Copy and paste relevant sections from the manuscript (include quotes in quotation marks "like this" to indicate direct quotes from your manuscript), or elaborate on this item by providing additional information not in the ms, or briefly explain why the item is not applicable/relevant for your study

您的回答

20) Trial limitations, addressing sources of potential bias, imprecision, and, if relevant, multiplicity of analyses

您的回复过长。请尝试让您的部分回答更简短。

20-i) Typical limitations in ehealth trials

Typical limitations in ehealth trials: Participants in ehealth trials are rarely blinded. Ehealth trials often look at a multiplicity of outcomes, increasing risk for a Type I error. Discuss biases due to non-use of the intervention/usability issues, biases through informed consent procedures, unexpected events.

	1	2	3	4	5	
subitem not at all important	○	○	○	○	○	essential

Does your paper address subitem 20-i? *

Copy and paste relevant sections from the manuscript (include quotes in quotation marks "like this" to indicate direct quotes from your manuscript), or elaborate on this item by providing additional information not in the ms, or briefly explain why the item is not applicable/relevant for your study

Like this:"This study has several limitations. Firstly, we were unable to implement blinding for the personnel involved in hypertensive health behavior interventions. To mitigate this, we established standardized intervention procedures, provided intervention strategies tailored to different patient types, edited intervention guidance language based on BCTs, incorporated the aforementioned content into the intervention manual, and conducted training for all intervention personnel. Secondly, the generalizability of our trial's results may be limited to hypertensive patient populations residing elsewhere, as they may possess distinct sociodemographic and comorbidity characteristics compared to our study participants. Thirdly, some measurement indicators relied on patient self-reports, which could potentially affect the credibility of the results. Fourthly, considering the workload, this study did not assess changes in compliance indicators in the control group, nor did it collect more comprehensive dietary-related information. This requires correction in future experiments of app-based hypertension health behavior interventions."

21) Generalisability (external validity, applicability) of the trial findings

NPT: External validity of the trial findings according to the intervention, comparators, patients, and care providers or centers involved in the trial

您的回复过长。请尝试让您的部分回答更简短。

21-i) Generalizability to other populations

Generalizability to other populations: In particular, discuss generalizability to a general Internet population, outside of a RCT setting, and general patient population, including applicability of the study results for other organizations

	1	2	3	4	5	
subitem not at all important	○	○	○	○	○	essential

Does your paper address subitem 21-i?

Copy and paste relevant sections from the manuscript (include quotes in quotation marks "like this" to indicate direct quotes from your manuscript), or elaborate on this item by providing additional information not in the ms, or briefly explain why the item is not applicable/relevant for your study

您的回答

21-ii) Discuss if there were elements in the RCT that would be different in a routine application setting

Discuss if there were elements in the RCT that would be different in a routine application setting (e.g., prompts/reminders, more human involvement, training sessions or other co-interventions) and what impact the omission of these elements could have on use, adoption, or outcomes if the intervention is applied outside of a RCT setting.

	1	2	3	4	5	
subitem not at all important	○	○	○	○	○	essential

Does your paper address subitem 21-ii?

Copy and paste relevant sections from the manuscript (include quotes in quotation marks "like this" to indicate direct quotes from your manuscript), or elaborate on this item by providing additional information not in the ms, or briefly explain why the item is not applicable/relevant for your study

您的回答

您的回复过长。请尝试让您的部分回答更简短。

OTHER INFORMATION

23) Registration number and name of trial registry

Does your paper address CONSORT subitem 23? *

Copy and paste relevant sections from the manuscript (include quotes in quotation marks "like this" to indicate direct quotes from your manuscript), or elaborate on this item by providing additional information not in the ms, or briefly explain why the item is not applicable/relevant for your study

Like this:"Trial registration number: ChiCTR2200062643 (Chinese Clinical Trial Registry, http://www.chictr.org.cn, registered on 14 August 2022)."

24) Where the full trial protocol can be accessed, if available

Does your paper address CONSORT subitem 24? *

Cite a Multimedia Appendix, other reference, or copy and paste relevant sections from the manuscript (include quotes in quotation marks "like this" to indicate direct quotes from your manuscript), or elaborate on this item by providing additional information not in the ms, or briefly explain why the item is not applicable/relevant for your study

Like this:"Sun T, Zhao H, Ding Z, Xie H, Ma L, Zhang Y, Wang Y, Yang Y, Xu C, Sun Y, Xu X, Ma Z. Evaluating a WeChat-Based Health Behavioral Digital Intervention for Patients With Hypertension: Protocol for a Randomized Controlled Trial. JMIR research protocols 2023 Sep 12;12:e46883. PMID:37698909"

25) Sources of funding and other support (such as supply of drugs), role of funders

您的回复过长。请尝试让您的部分回答更简短。

Does your paper address CONSORT subitem 25? *

Copy and paste relevant sections from the manuscript (include quotes in quotation marks "like this" to indicate direct quotes from your manuscript), or elaborate on this item by providing additional information not in the ms, or briefly explain why the item is not applicable/relevant for your study

Like this:"This work was supported by the Demonstration of Integrated Application of Community Science and Fitness in the Central Region (grant 2022YFC2010200), Anhui Provincial Department of Science and Technology (grant 202103a07020004) and the Anhui Provincial Department of Education (grant SK2021A0432, 2022lhpysfjd063 and gxyq2022040). The funding source had no role in the study design, data collection, management, data analysis, data interpretation, report writing, or the decision to submit for publication. The authors would like to thank Huaqing Liu and Lihua Zhou for preparing the simplified DASH grading diet index score."

X27) Conflicts of Interest (not a CONSORT item)

X27-i) State the relation of the study team towards the system being evaluated

In addition to the usual declaration of interests (financial or otherwise), also state the relation of the study team towards the system being evaluated, i.e., state if the authors/evaluators are distinct from or identical with the developers/sponsors of the intervention.

	1	2	3	4	5	
subitem not at all important	○	○	○	○	○	essential

Does your paper address subitem X27-i?

Copy and paste relevant sections from the manuscript (include quotes in quotation marks "like this" to indicate direct quotes from your manuscript), or elaborate on this item by providing additional information not in the ms, or briefly explain why the item is not applicable/relevant for your study

您的回答

您的回复过长。请尝试让您的部分回答更简短。

As a result of using this checklist, did you make changes in your manuscript? *

- (selected) yes, major changes
- yes, minor changes
- no

What were the most important changes you made as a result of using this checklist?

您的回答

How much time did you spend on going through the checklist INCLUDING making changes in your manuscript *

I spent two weeks checking the list, including changes made to the manuscript.

As a result of using this checklist, do you think your manuscript has improved? *

- (selected) yes
- no
- 其他:

Would you like to become involved in the CONSORT EHEALTH group?

This would involve for example becoming involved in participating in a workshop and writing an "Explanation and Elaboration" document

- yes
- no

您的回复过长。请尝试让您的部分回答更简短。

Any other comments or questions on CONSORT EHEALTH

您的回答

STOP - Save this form as PDF before you click submit
To generate a record that you filled in this form, we recommend to generate a PDF of this page (on a Mac, simply select "print" and then select "print as PDF") before you submit it.

When you submit your (revised) paper to JMIR, please upload the PDF as supplementary file.

Don't worry if some text in the textboxes is cut off, as we still have the complete information in our database. Thank you!

Final step: Click submit !
Click submit so we have your answers in our database!

提交 清除表单内容

切勿通过 Google 表单提交密码。

此内容不是由 Google 所创建，Google 不对其作任何担保。 举报滥用行为 - 服务条款 - 隐私权政策

Google 表单

您的回复过长。请尝试让您的部分回答更简短。

您的回复过长。请尝试让您的部分回答更简短。
